# The 20th Biennial congress of the South African Society of Nuclear Medicine conference report

**DOI:** 10.4102/jcmsa.v1i1.55

**Published:** 2023-12-22

**Authors:** Kgomotso M.G. Mokoala

**Affiliations:** 1 Specialist Nuclear Physician, Head of Clinical Unit, Department of Nuclear Medicine, Steve Biko Academic Hospital, University of Pretoria, Pretoria, South Africa

## Abstract

**Sponsor disclaimer:** We would like to acknowledge our generous sponsor, Axim Nuclear and Oncology (Pty) Ltd (https://www.axim.co.za), which made the publication of the conference report possible.

## Introduction

The South African Society of Nuclear Medicine (SASNM), which was founded in 1974 is one of the oldest nuclear medicine societies and probably the most active on the African continent. South African Society of Nuclear Medicine has approximately 200 members, including nuclear medicine physicians, radiographers, physicists, radiopharmacists and scientists. The SASNM recently held its 20th iteration of the Biennial Conference in the lovely city of Gqeberha from 24 August 2023 to 27 August 2023. The overarching theme of the congress was ‘Going back to our roots’, which is very relevant given the global theragnostics developments and it embodied four main pillars, namely, the evolution of nuclear medicine, focus on women in nuclear medicine, focus on basic nuclear medicine imaging and procedures as well as a focus on the referring clinicians. The meeting was a great success and had world-renowned speakers from five continents. We had an overwhelming influx of excellent abstracts and 56 of those we accepted and presented at the meeting and in this publication.

This meeting reflected the tremendous growth in theragnostic: an amalgamation of the words therapeutics and diagnostics, which simply put means ‘see it, treat it’. It bears testament to the vision of Dr Saul Hertz who first performed therapy for thyroid disorder in 1941.^1^ South Africa is one of the world leaders in this area of targeted radionuclide therapy especially using alpha emitters.

The conference also addressed the lack of female representation in science leadership positions including editorial boards of radiology and nuclear medicine journals.^2^ This is testament to the amount of work that still needs to be performed as women in order to gain recognition for our contribution. This was the motivation for our first ever Women’s breakfast symposium sponsored by the South African Nuclear Medicine Research Infrastructure (NuMeRI) wherein we had a panel discussion with important local and international female leaders addressing issues around capacitating women and mentorship.

While South Africa is a global player in the Nuclear Medicine field, as it is recognised and has been selected as the highlight county in the SNMMI 2024 conference in Toronto, we cannot ignore the rest of the African continent. Nuclear medicine is an ever-evolving field and often times with the latest developments we find that middle- to low-income countries lag behind. Hence, the meeting highlighted the fact that as South Africa in collaboration with the International Atomic Energy Agency (IAEA) we should continue to train fellows from all over Africa in various nuclear-related fields. We also need to formulate guidelines based on our own data and local circumstances.

Lastly, our field relies on referrals from the clinicians from various specialties. We felt it was important to invite clinicians to engage them on areas in which we are doing well and also on areas we can improve on to better assist them with patient management. We also need to engage in more multidisciplinary (MDT) discussions as our roles as nuclear physicians evolve into therapy:

‘Its in the **roots,** not the branches, that a trees greatest strength lies.

If you know where you are from, it is harder for people to stop you where you are going.

A tree’s beauty lies in its branches, but its strength lies in its **roots.**’

We hope that it is in the going back to our roots that we find direction for our future. That future we hope to be patient orientated in our approach, we hope to have more clinical trials coming from us to produce good quality and highest evidence and lastly, we hope to foster collaborations both with clinicians and with fellow nuclear physicians locally and abroad.

## Conference Abstracts

### Evaluation and development of quality control procedures for technetium-99m labelled macro aggregated albumin at a tertiary hospital in Gauteng

**L. Amondi^1^, M. Malope^2^, M. Lundie^3^, L. Mosima^4^**

^1^ *Sefako Makgatho Health Sciences University, Pretoria, South Africa*

^2^ *Dr. George Mukhari Academic Hospital, Pretoria, South Africa*

^3^ *Sefako Makgatho Health Sciences University, Pretoria, South Africa*

^4^ *Sefako Makgatho Health Sciences University, Pretoria, South Africa*

#### Background

The radiopharmaceutical, technetium-99m labelled macro aggregated albumin (99mTc-MAA) is an injectable diagnostic agent used in nuclear medicine for lung perfusion imaging in patients with suspected pulmonary embolism.

#### Aim

Ventilation and lung perfusion imaging scans (V/Q scans) are performed routinely in the Nuclear Medicine Department at Dr. George Mukhari Academic Hospital (DGMAH) to assess patients with suspected pulmonary embolism.

#### Methods

Little documented information exists about the quality control procedures of 99mTc-MAA for lung perfusion imaging at the tertiary hospital. The need for evaluation and development of the quality control procedures is based on establishment of a new single photon emission computed tomography (SPECT) unit at the tertiary hospital in 2019. In practice, when validated quality control procedures such as those in the pharmacopoeias and in the accepted guidelines are impractical to perform in a hospital radiopharmacy because of various reasons such as technical complexity of the procedures, expensive analytical components, time consumption, toxicity of solvents, alternative quality control procedures can be developed that are faster, simplified and cost effective. For this reason, 99mTc-MAA is one of the radiopharmaceuticals currently being used at the hospital; hence, there is an existing need for evaluation and development of quality control procedures for the radiopharmaceutical.

#### Results

The main objective of this study is to validate current quality control processes for 99mTc-MAA at the tertiary hospital’s Nuclear Medicine Department, to identify any missing or undetermined quality control processes and to develop these quality control procedures according to the relevant guidelines. The research methodology is quantitative, analytical and experimental. The methods involve experimental tests on the radiopharmaceutical and these are: radionuclidic purity, radiochemical purity, particle size, particle number and pH test. The outcome of this study is to develop a quality control tool for 99mTc-MAA where necessary and to validate the existing quality control procedures on the radiopharmaceutical.

#### Conclusion

The implication of this study to other researchers is the provision of a validation method for quality control procedures of radiopharmaceuticals. Newly set-up hospitals can use the validation methods in this study, as well as alternative quality control procedures that this study seeks to develop.

#### Keywords

Technetium-99m labelled macroaggregated albumin, validation, quality control tool, development, quality control procedures.

### Validation of a gas chromatography method for the determination of residual solvents in positron emission tomography radiopharmaceuticals

**B. Adedeji^1^, J. le Roux^1^, S. Rubow^1^**

^1^ *Stellenbosch University, Cape Town, South Africa*

#### Background

Positron emission tomography (PET) radionuclides have a short half-life and, unlike conventional pharmaceuticals, must usually be prepared, tested, and administered to patients within a very short time. The current European Pharmacopeia (EP) method for determining residual solvents using gas chromatography flame ionisation detection (GC-FID) is tedious and time-consuming. Apart from a long analysis time of ± 60 min, some peaks were not well defined on chromatograms and peak tailing occurred. The validation of non-pharmacopeial analytical procedures is necessary to demonstrate that they are fit for the intended purpose.

#### Aim

The purpose of this research was to validate a much faster in-house developed method, using the International Conference on Harmonisation for Technical Requirements for Registration of Pharmaceuticals for Human Use (ICH) guidance document.

#### Methods

Standard solutions were prepared in-house using the highest grade ethanol, methanol and acetonitrile. The method was then validated in accordance with the ICH guidelines. Parameters that were tested included specificity, precision, accuracy, linearity, range, limit of detection (LOD), limit of quantification (LOQ) and robustness. Linearity was established over a concentration range of 50% to 150% of the ICH limits for all the solvents.

#### Results

The in-house developed method had a total analysis time of 8 min, which is significantly shorter than the method described in the EP. The method was also found to be specific, accurate, precise and linear. The method was able to detect and quantify all the residual solvents present in 18F-fluorodeoxyglucose ([18F]FDG) and [18F] FMISO samples. The LOD and LOQ were found to be 75 ppm and 750 ppm for methanol; 250 ppm and 1000 ppm for ethanol; 20.5 ppm and 102.5 ppm for acetonitrile, respectively. The method was also found to be precise, with intra-day and inter-day relative standard deviations (RSDs) of less than 3%. Accuracy was determined by recovery of the samples, ranging from 99.5% to 99.7%.

#### Conclusion

An in-house method for determining residual solvents in PET radiopharmaceuticals was successfully tested and validated using ICH guidelines. The results showed that this method decreased the analysis time significantly and can be used on a routine basis.

### The influence of low-dose computed tomography attenuation correction on artefacts of myocardial single photon computed tomography images for nuclear medicine studies

**L. Nolan^1,2^, N. Korowlay^1^, M. Morkel^3^, C. Lackay^2^, M. du Toit^1^, E. Seane^2^**

^1^ *Nuclear Medicine, Tygerberg Hospital, Parow valley, Cape Town, South Africa*

^2^ *Cape Peninsula University of Technology, Bellville, Cape Town, South Africa*

^3^ *Dr HR Morkel inc. Panorama Hospital, Panorama, Cape Town, South Africa*

#### Introduction

Artefacts in myocardial perfusion single photon emission computed tomography (SPECT) imaging cause poor image quality, which may present as false-positive results that may lead to unnecessary, expensive and invasive procedures. Breast and diaphragm attenuation may cause anterior and/or inferior soft tissue artefacts and may interfere with the visualisation of the perfusion defects of the myocardium. The myocardial perfusion studies were performed with Tc-99m sestamibi, comprised of stress/rest images with patients positioned in a supine or prone position.

#### Aim

The aim of the study was to determine whether hybrid imaging, SPECT with computed tomography (CT), will improve the image quality by reducing the soft tissue artefacts with the application of attenuation correction maps in the stress and rest Tc-99m sestamibi myocardial SPECT perfusion studies.

#### Materials and Methods

A retrospective study was performed on 100 image data sets of patients with suspected ischaemic heart disease referred to the Nuclear Medicine Department. The no attenuation corrected (NAC) and the attenuation corrected (AC) stress and rest images were the dependent variables of the study. The independent variables were the relevant factors such as gender, age, weight, height and position of the patients during imaging were also reviewed for their influence on the attenuation correction of the images. The results of the patients were quantitatively reviewed and the images were given a score of 0–4. An overall score of the results of the improvement (yes) versus no improvement (no) of attenuation correction application (AC-A) was also given. The critical assessment areas were the inferior and anterior wall defects and analysis was performed by the two nuclear medicine physicians. A generalised linear model was applied to determine relationships between the dependent and independent variables.

#### Results and Discussion

The effect of the independent variable on the dependent variable with a *p*-value of > 0.05, indicated non-significance. The results demonstrated that with anterior stress and rest SPECT images, the most artefacts were created with AC-A. Improvement for the stress inferior images was 61%. Attenuation corrected-A to the stress and rest AC and NAC images scored an improvement of 49%. Although there was no improvement of 51% for the overall outcome of stress/rest studies, it included 18 stress studies that had improvement and nine rest studies improved (scores 2–3) in the inferior wall of the myocardium.

#### Conclusion

The AC-A scores of the images for AC versus NAC with MPI showed a significant influence on the outcome of the study on the inferior wall of the myocardium.

### Radiolabelling of chelators DOTA, EDTA and DTPA with gallium-68

**J. Van Zyl^1,2^, J. le Roux^3^, S. Rubow^1^, J. Kleynhans^4^**

^1^ *Stellenbosch University, Cape Town, South Africa*

^2^ *Axim Nuclear and Oncology, Johannesburg, South Africa*

^3^ *Nuclear Medicine Research Infrastructure Node of Infection Imaging, Cape Town, South Africa*

^4^ *Laboratory for Radiopharmaceutical Research, Leuven, Belgium*

#### Introduction

The use of [68Ga]Ga-EDTA, [68Ga]Ga-DTPA and [68Ga]Ga-DOTA in cisternography has been described. If such products are to be used in a clinical setting, reliable production and quality control methods are essential.

#### Aim

The aim of this study was to validate iTLC and high-performance liquid chromatography (HPLC) methods to determine radiochemical purity of selected chelators and to translate manual methods into a suitable automated radiolabelling method.

#### Methods

Manual radiolabelling of chelators DOTA, EDTA and DTPA with gallium-68 was performed as previously described. Gallium-68 was obtained from an iThemba LABS germanium-68/gallium-68 generator less than 1 year old using pre-purification or fractionated elution. Labelling was performed at pH 4.5; incubated at 95°C for 10 min. Solid-phase extraction was used to remove colloids from the final product whereafter a quality control sample was obtained for iTLC and HPLC analysis. A two-strip SG-iTLC method was validated with a range of concentrations using acetonitrile and TFA as mobile phases. A radio-HPLC method was developed (Phenomenex Jupiter 4 µm Proteo 90A Column, 250 mm × 4.6 mm) using gradient elution conditions with acetonitrile and deionised water and flow rate 1 ml/min. The optimal labelling parameters determined by manual synthesis were translated to an automated synthesis method using the Scintomics GRP 3V module.

#### Results

Fractional elution or pre-purification followed by 1000 µL of sodium chloride elution resulted in the highest concentration of Ga-68 for radiolabelling of chelators. iTLC analysis showed free Ga-68 at solvent front (Rf = 0.4–0.6) in TFA whereas free gallium-68 and colloid remained at the origin (Rf = 0) in acetonitrile. A solvent mixture of 50% acetonitrile and 4% TFA resulted in optimal peak resolution for [68Ga]Ga -EDTA and [68Ga]Ga -DOTA whereas for [68Ga]Ga -DTPA the solvent mixture consisted of 20% acetonitrile and 4% TFA. The HPLC analysis revealed a retention time of 4.34–4.58 min for both free gallium-68 and radiolabelled 68Ga-chelators with no other radiochemical impurities present. Automated radiolabelling of [68Ga]Ga -DOTA showed a radiochemical purity > 95% on ITLC analysis.

#### Conclusion

The optimised radio-iTLC method showed separation of colloids, free gallium-68 and radiolabelled chelators. The HPLC analysis may require a different selection of stationary and mobile phases for separation of radiochemical species. Formulation requirements relevant to intrathecal administration of radiopharmaceuticals will be addressed in future studies.

### Dacryoscintigraphy in a traumatic lacrimal duct injury

**W. Endres^1^, G. Engelbrecht^1^, O. Evboumwan^1^, T. Tebeila^1^**

^1^ *University of the Free State, Bloemfontein, South Africa*

#### Introduction

Epiphora may be secondary to problems associated with secretion or excretion of the lacrimal system. It is important to know which of these problems are associated with a patient’s presentation, as the management of both problems are different. Ultrasonography can be used to produce images of the lacrimal sack and the flow of tears; however, the ultrasonic probe exerts pressure on the medial canthal area (potentially affecting pressure gradients created under physiological conditions). Dynamic MRI studies of lacrimal drainage are possible but challenging to perform. Dacryoscintigraphy, a non-invasive, functional examination of the lacrimal system uses technetium-99m pertechnetate to evaluate tear flow through the lacrimal drainage system. Nasolacrimal duct obstruction is a common problem, especially in children. Trauma to the canaliculus most often leads to epiphora. Repair of the canaliculus lessens the risk of the patient developing epiphora. Dacryoscintigraphy offers the referral doctor the opportunity of knowing if the epiphora is because of an obstruction, and it can further determine at what level the obstruction has taken place. It has been extensively used to facilitate the definite diagnosis of obstructions and stenosis of the lacrimal drainage system. Dacryoscintigraphy is easy to perform and is non-invasive and this is an advantageover contrast dacryocystography, thus avoiding general anaesthesia in children.

#### Methods

A dacryoscintigraphy was performed on a 7-year-old patient who sustained a dog bite injury to the face 2 years prior, and now presenting with right eye epiphora. The patient received 1 drop of 100uCi Tc-99 Pertechnetate to the lateral canthus of each eye. Dynamic images followed by serial static images were acquired.

#### Result

In the left lacrimal system, immediate visualisation of the lacrimal sac, with spontaneous drainage into the lacrimal duct was observed. There was progressive accumulation of the radiotracer in the lacrimal sac of the injured right eye, up to the end of the study, without any notable drainage into the nasolacrimal duct noticed. The study concluded that there was evidence of obstruction at the level of the left lacrimal sac.

#### Conclusion

Dacryoscintigraphy mimics ‘physiological’ lacrimal outflow and can be performed under normal pressure gradients, it is considered more suitable for the study of functional epiphora. In this clinical case it confirmed that the epiphora was because of an obstruction and this was able to guide the next appropriate management in this patient.

### Can FDG PET/CT be used to optimise the treatment of patients with pulmonary tuberculosis?

**A. Ismaila^1^, I.O. Lawal^1,2^, G.O. Popoola^3^, M. Mathebula^4^, I. Moagi^4^, K. Mokoala^1^, N. Moeketsi^4^, M. Nchabeleng^4^, C. Hikuam^5^, M. Hatherill^5^, B.P. Fourie^6^, M.M. Sathekge^1,7^**

^1^ *Department of Nuclear Medicine, University of Pretoria, Pretoria, South Africa*

^2^ *Department of Radiology and Imaging Sciences, Emory University, Atlanta, GA, United States of America*

^3^ *Saxon Court Lincolnshire Partnership NHS foundation trust (LPFT), Lincoln, United Kingdom*

^4^ *Department of Medical Microbiology and MeCRU, Sefako Makgatho University of Medical Science, Pretoria, South Africa*

^5^ *South African Tuberculosis Vaccine Initiative, Institute of Infectious Disease and Division of Immunology, Department of Pathology, University of Cape Town, Cape Town, South Africa*

^6^ *Department of Medical Microbiology, University of Pretoria, Pretoria, South Africa*

^7^ *Nuclear Medicine Research Infrastructure (NuMeRI), Steve Biko Academic Hospital, Pretoria, South Africa*

#### Background

The treatment of pulmonary tuberculosis is long because of the slow-growing nature of *Mycobacterium tuberculosis* (MTb). At the end of a standard course of anti-tuberculous treatment (ATT), some patients still harbour slow-growing MTb (sgMTb) in their lesions, which are responsible for relapse. These sgMTb are non-culturable and are difficult to detect. We investigate the utility of end-of-treatment 18F-fluorodeoxyglucose ([18F]F-FDG) positron emission tomography/computed tomography (PET/CT) to differentiate patients with sterilising cure versus those with sgMTb who are at risk for relapse.

#### Methods

We prospectively recruited patients who had a standard course of ATT. All patients underwent [18F]F-FDG PET/CT within 2 weeks of completing ATT. We determined the presence of residual metabolic activity (RMA), which is FDG uptake in the lungs above mediastinal background activity on the [18F]F-FDG PET/CT. Patients were classified as having RMA or complete metabolic response (CMR) to ATT. All patients were subsequently followed-up after completing [18F]F-FDG PET/CT imaging for relapse. Confirmatory bacteriologic testing was performed in patients with clinical suspicion of relapse. In those who relapsed, a repeat FDG PET/CT imaging was subsequently performed.

#### Results

We studied 75 patients including 50 HIV-infected individuals with a mean age of 36.09–10.49. The median CD4 count among HIV-infected patients was 255 cells/mm^3^ (IQR: 147–448). The HIV viral load was 12 497 copies/mL (IQR: 158-38841). The HIV-infected patients had lower haemoglobin levels (13.07 g/dL ± 1.78 g/dL vs. 14.24 ± 2.07, *p* = 0.021) and higher C-reactive protein levels (5.70 vs. 1.20, *p* = 0.001) compared with HIV-uninfected patients. All other baseline clinical and demographic characteristics were not significantly different between the groups. Forty-one patients had RMA and its incidence was not significantly different between HIV-infected and uninfected patients (*p* = 0.101). Thirty-four patients demonstrated complete metabolic response (CMR) to ATT. No tuberculosis relapse was demonstrated in those with CMR. In the RMA group, three patients (7.3%) relapsed on follow-up. Relapse was confirmed bacteriologically in all the three patients. Repeat [18F]F-FDG PET/CT imaging at the time of clinical relapse demonstrated the persistence of RMA in the same lung regions in all three patients.

#### Conclusion

Fluorodeoxyglucose PET/CT imaging demonstrates a high prevalence of RMA among patients treated with a standard course of ATT for PTB, with a similar incidence between HIV-infected and uninfected patients. Complete metabolic response on post-treatment [18F]F-FDG PET/CT is consistent with sterilising a cure for pulmonary tuberculosis. Patients with RMA on post-treatment [18F]F-FDG PET/CT are at risk of tuberculosis relapse. These results show the potential of [18F]F-FDG PET/CT for use as a non-invasive biomarker for drug development and in investigating shorter ATT regimens.

### Radiopharmacy services in South African academic hospitals and implications for planning new services

**D. Kimani^1^, B. Summers^1^, L. Mosima^1^**

^1^ *Sefako Makgatho Health Sciences University, Ga-Rankuwa, South Africa*

#### Introduction

Nuclear medicine (NM) plays an important role in diagnosis and treatment of a wide range of diseases. Optimal radiopharmacy services are key to progress in both patient care and research. Underdeveloped countries are embracing NM as a speciality. To establish new NM and radiopharmacy centres, proper planning is essential because of the high costs of staffing, equipment and radiopharmaceuticals. This study describes the pattern of radiopharmacy services and staffing in NM departments in South African Academic Hospitals (SAAHs); this information can help plan new nuclear and radiopharmacy centres in similar settings.

#### Objectives

The primary objective was to determine the utilisation of radiopharmacy services in NM departments in SAAHs and to develop a tool to help plan new radiopharmacy centres in African settings.

#### Method

The study was retrospective (2022), descriptive and quantitative with qualitative aspects. An anonymised questionnaire was sent via SurveyMonkey^®^ to the nine SAAHs, to determine hospital and specialist unit sizes, NM patient numbers by scan type, identify facilities (cameras and equipment), staffing levels, commonly used radiopharmaceuticals and to define basic requirements for establishing new facilities.

#### Results

The initial response rate was 67% (6/9 hospitals). Preliminary results: Hospital sizes ranged from 846 to 3200 beds, average number of NM patients seen was 3526 for 2022. All hospitals have single photon computed emission tomography/computed tomography (SPECT/CT) cameras; five have positron emission tomography – computed tomography (PET-CT) cameras and one refers positron emission tomography (PET) patients to a private hospital. Only two hospitals have hot cells. All have NM physicians (range 3–13, average 5) and at least one medical physicist (range 1–4) plus nuclear medicine technologists (range 8–15, average 9). Only two hospitals have radiopharmacists (one each) and they also have hot cells. Staff, equipment, patient load and radiopharmaceuticals prepared will be related to patient specialist unit sizes and to catchment population to provide guidelines for start-up services.

#### Conclusion

To promote optimal patient services and support research in SAAHs, norms must be developed that are applicable to Africa. The two hospitals with hot cells have radiopharmacists, indicating these staff are key to progress. The study’s findings will be compared with International Atomic Energy Agency staffing norms to provide recommendations for African settings. Appropriate staff quotas and equipment are required to promote optimal use of radiopharmaceuticals and enable the NM team to operate to their full potential.

### Radiopharmaceuticals analysis: Biomedical chromatography applications

**S. Mdanda^1^**

^1^ *Nuclear Medicine Research Infrastructure NTP, Nuclear Medicine Department, South Africa*

Radiopharmaceutical synthesis methods use relatively large amounts of reagents, necessitating the use of high-resolution and high-capacity chromatographic methods. Furthermore, purification is required prior to the formulation of radiopharmaceuticals for injection to remove various impurities. However, for routine injectable production, radio-thin layer chromatography (i-TLC) is preferred because of short time runs and user friendliness. The problem with i-TLC is that TCL plates are not as efficient as high-performance liquid chromatography (HPLC) columns and are also limited to radioactive detection, so impurities are frequently missed. In this study, the aim was to develop and validate shorter liquid chromatography methods utilising Gabi, a diode array (UV-VIS), and an evaporative light scattering detector (ELSD) all in conjunction. The analytes of interest in this study were 68Ga- Fapi, 68Ga-Dotatate, and 68Ga-prostate-specific membrane antigen-617. Degradation of chelators and the introduction of impurities can occur during radiopharmaceutical synthesis. If the sample contains chromophores, the UV-VIS detector can detect them all. The Gabi is capable of detecting all substances containing radionuclides that emit gamma rays. All substances that can diffract light can be detected by the ELSD detector. In this study, it was demonstrated that a simple (5–10 min) HPLC analytical method with three simultaneous detection systems can be more profitable than the conventional iTLC method for determining the purity of radiopharmaceuticals.

### Visual and quantitative comparison of different imaging reconstructions on a clinical acquisition

**L. Smith^1^, C. Swanepoel^1^, H. Ndlovu^1,2^, K. Ramonaheng^1^, T. Ebenhan^1,2,3^, M. Sathekge^1,2^**

^1^ *NuMeRI NPC, South Africa*

^2^ *University of Pretoria, Pretoria, South Africa*

^3^ *South Africa Nuclear Energy Corporation, Hartbeespoort, South Africa*

#### Background

Filtered backprojection (FBP) is the most basic image reconstruction algorithm used for positron emission tomography (PET). It is also the algorithm of choice to compare acquisition quality between vendors. Advances in system hardware and software now allow for various processing algorithms and applications to be used. These include iterative algorithms (ordered subset expectation maximisation [OSEM]/MOSEM), point-spread-function (PSF) and time of flight (TOF) applications. Each of these applications can affect quantifiable data obtained from clinical acquisitions. Showcasing the quantitative differences using alternate reconstruction algorithms while viewing 18F-fluorodeoxyglucose (18F-FDG) will benefit troubleshooting in a clinical setting and highlight the importance of consistent image reconstruction.

#### Methodology

Retrospective patient data acquired on a Siemens Biograph Vision 450 positron emission tomography/computed tomography (PET/CT) scanner, with a 64-slice computed tomography (CT), was used. Four (*n* = 4) 18F-FDG scans were reconstructed using five reconstruction techniques (FBP, OSEM with and without TOF, MOSEM with and without TOF), with CT attenuation correction. Standard uptake value (SUVmean) for target organs (brain, heart, liver) was obtained as well as SUVmean for blood pool background (superior vena cava). Target organ to blood pool ratio was obtained (SUVR) and averaged over the four acquisitions for each of the five reconstruction methods.

#### Expected findings and conclusion

As expected, differences in PET-CT reconstruction influenced factors such as SUV values (or SUVR) and effects visual changes. Therefore, it would be advised to identify an optimal reconstruction method and apply it to all scans acquired at a given facility as it also influenced the visual assessment of the images by the physicians.

In conclusion, choosing suitable reconstruction parameters is critical for producing high-quality PET- CT images. A comprehensive understanding of the influence of these parameters on image quality can aid in optimising the imaging process, ultimately improving diagnostic accuracy.

### Developing a radiolabelled tracer for prospective PET imaging of *Plasmodium falciparum* infection

**J. Duvenhage^1^, J. Zeevaart^1,2,3,4^, T. Ebenhan^1,2,3,4^**

^1^ *Nuclear Medicine Research Infrastructure, Pretoria, South Africa*

^2^ *Radiochemistry, The South African Nuclear Energy Corporation, Pelindaba, South Africa*

^3^ *DSI/Preclinical Drug Development Platform, North-West University, Potchefstroom, South Africa*

^4^ *Department of Nuclear Medicine, University of Pretoria, Pretoria, South Africa*

#### Background

*Plasmodium falciparum* (*P. falciparum*) causes the most severe cases of malaria. Severe malaria (SM), an understudied multisystem disease, affects the host’s organs and can lead to serious complications, some effecting life-long neurological and cognitive sequela. A major limitation of understanding SM is the inability to visualise the host-parasite mechanisms governing pathogenicity in situ. Nuclear imaging could aid in investigating malaria-related pathologies; however, there are no malaria-specific tracers. Therefore, we aimed to develop zirconium-89 (89Zr) and gallium-68 (68Ga)-radiolabelled malaria-specific tracers, assess their stability, in vitro characteristics and in vivo biodistribution using non-invasive micro positron emission tomography/computed tomography (microPET/CT) imaging.

#### Methods

A plasmodium-specific antibody (IIIB6), antibody fragment (Pf-Fab) and peptide (P1) were investigated. IIIB6 and Pf-Fab were conjugated to p-isothiocyanatobenzyl-desferrioxamine followed by complexation of 89Zr. [89Zr]Zr-IIIB6 and [89Zr]Zr-Pf-Fab were injected into healthy BALB/c mice and assessed by microPET/CT imaging and post-mortem biodistribution. A 1,4,7,10- tetraazacyclododecane-1,4,7,10-tetraacetic acid (DOTA)-Gly-functionalised P1 derivative (DOTA-P1) underwent 68Ga-radiolabelling. Radiolabelling was optimised by adjusting radiolabelling reaction conditions. Radiolabelling efficiency (LE), radiochemical yield and stability were assessed with radio-HPLC and iTLC. In vitro studies for [68Ga]Ga-DOTA-P1 included plasma stability, red blood cell (RBC) binding and plasma-protein binding.

#### Results

Quantification of the microPET/CT image-guided cardiac region over 24 h indicated pharmacological half-lives of 7.1–12.1 h and 3.5–6.0 h for [89Zr]Zr-IIIB6 and [89Zr[Zr-Pf-Fab, respectively. Unfavourably-high concentration of [89Zr]Zr-IIIB6 in liver occurred after 2–6 h and moderate spleen>kidneys>heart>stomach>lung>femur uptake. For [89Zr[Zr-Pf-Fab, unwanted kidney uptake occurred after 4–6 h, and moderate uptake in liver>lung>stomach>femur. Ex vivo biodistribution studies confirmed high thermodynamic and physiological stability of both tracers and no organ toxicity. DOTA-P1 was successfully radiolabelled with 68Ga and purified yielding a radiochemical purity exceeding 97%. Efficient preparations (including purification) of [68Ga]Ga- DOTA-P1 (LE = 77% – 99%) at a concentration of 22.7 nmoL/mL was achieved after 30 min incubation at 95°C. This optimised radiosynthesis achieved a radiochemically pure product (> 99%) of adequate molar activities (> 1.0 GBq/µmoL) for preclinical application. [68Ga]Ga-DOTA-P1 maintained stability in human plasma up to 2 h (> 90%), showed moderate RBC association (± 25%) and high serum protein binding (± 87%).

#### Conclusion

The development of three radiotracers for future malaria-specific imaging is presented. The results support that [89Zr]Zr-Pf-Fab has the more preferable pharmacological profile over [89Zr]Zr-IIIB6 and is suggested for future investigation in *P. falciparum*-infected animals. Further tests of [89Zr]Zr-Pf-Fab should include co-administrations of compounds that may reduce its unwanted kidney retention. The robust radiolabelling protocol along with its high serum stability and low-level RBC binding supports future pre-clinical characterisation of [68Ga]Ga-DOTA-P1 in vivo.

### Radiation exposure of nuclear medicine radiographers and radiopharmacists during their PET-CT rotation

**W. Pilloy^1^, T. Mabhengu^2^, N. Nyakale^1^**

^1^ *SMU Nuclear Medicine*

^2^ *SMU Medical Physics*

#### Purpose

Radiographers and radiopharmacists working at a positron emission tomography computed tomography (PET CT) facility are among the professions most exposed to radiation in nuclear medicine. This study assessed the exposure of our staff during a period of six consecutive months to evaluate their exposure per day and per examination and to compare it with published data.

#### Material and method

Seven direct reading dosimeters, by different suppliers, were issued to eight radiographers, and three to five radiopharmacists. Qualified student professionals were involved. More than one radiographer and one radiopharmacist were working practically every day. Only vendor supplied 18F labelled tracers were used.

The following data were entered by each personnel on personalised article documents: date, person’s identity, dosimeter number, start reading, closing reading. After transcription on Excel, calculation of the individual daily dose and sorting by date were performed. Cross-correlation with the positron emission tomography (PET) database allowed the calculation of the dose per PET examination.

#### Results

There were 140 entries for radiographers and 112 entries for radiopharmacists.

In the case of radiographers, occasionally high exposures resulted in higher average exposure (28.5 uSv/day). When these peaks were excluded, the average exposure per patient was 17.5 uSv/day or 4 uSv/PET exam. Radiopharmacists had an average daily exposure of 13.7 uSv/day.

Several issues were noticed. The transcription of article data into Excel was time-consuming and prone to errors. The collection of data was irregular over the period of investigation. All dosimeters were not necessarily equally calibrated, with results either in milliSievert or in microSievert. Some personnel seemed more prone to higher exposure, although in rather exceptional circumstances.

#### Conclusion

The exposure recorded at our institution remained within the limits of the IRCP and compared favourably with the figures recorded by others.

Among the improvements suggested by this study, are: the direct entry of data in Excel, regular review of the readings, compliance to existing standard operating procedures, calibration of the dosimeters in the same units, supervision of junior staff, and appropriate shielding (portable screen or injection box) and/or an automated injector.

In addition, time of flight cameras and data recovery by artificial intelligence are designed to limit radiation exposure to both patients and staff and allow the recording of PET images with much lower activity.

### Synthesis, radiolabelling and preliminary in vitro assessment of novel radiopharmaceuticals as potential bacterial-specific radiotracers using positron emission tomography imaging

**C. Gouws^1^, J. Duvenhage^2,3^, S. Mdanda^3^, J. Zeevaart^2,3^, M. Sathekge^4^, T. Naicker^1^, H. Kruger^1^, T. Govender^5^, T. Ebenhan^2,3,4^**

^1^ *Catalysis and Peptide Research Unit, School of Health Sciences and School of Chemistry and Physics, University of KwaZulu-Natal, Durban, South Africa*

^2^ *Radiochemistry, the South African Nuclear Energy Corporation (Necsa) SOC Ltd, Pelindaba, South Africa*

^3^ *Nuclear Medicine Research Infrastructure NPC, Pretoria, South Africa*

^4^ *Department of Nuclear Medicine, University of Pretoria, Pretoria, South Africa*

^5^ *Department of Chemistry, University of Zululand, KwaDlangezwa, South Africa*

#### Background

The current positron emission tomography (PET)-radiopharmaceuticals used for diagnosis of bacterial infections rely on secondary host-mediated inflammatory responses, leading to false-positive findings because of misinterpreted, sterile inflammation. To discriminate between infection and inflammation, a more direct targeting mechanism for bacteria is desirable. Therefore, the development of bacteria- selective PET-radiotracers is a valid approach to improve differential diagnosis of bacterial infections and monitoring the performance of treatment interventions using non-invasive nuclear imaging. In this study three candidate molecules with known bacterial-specific interaction were deemed appropriate radiotracer vector scaffolds when functionalised with 1,4,7-triazacyclononane-1-succinic acid-4,7-diacetic acid (NODASA) to subsequently allow for gallium-68 complexation: (1) NODASA-D-lysine (NDL) and two *Mycobacterium tuberculosis* (MTB)-specific phage display-generated peptides also known as (2) NODASA-PH1 and (3) NODASA-H8.

#### Methods

Solid phase peptide synthesis (SPPS) was used to synthesise these compounds, including NODASA-functionalised L-lysine (NLL; negative control), followed by purification using reverse-phase preparative high-performance liquid chromatography (HPLC), and characterisation using HRMS/LCMS. Compounds were radiolabelled with generator-eluted 68Ga-activity, the radiosynthesis was optimised by adjusting reaction conditions such as pH, temperature and NODASA-compound concentration. Radiolabelling efficiency, radiochemical yield and radiochemical purity was assessed with radio-HPLC or TLC. The radio-metal complex stability was assessed using the EDTA trans-chelation challenge. In vitro studies included quantification of plasma stability, blood cell association, and plasma-protein binding assays to characterise the compound stability, radioisotope integrity and estimated bioavailability following intravenous administration into human blood as a potential PET radiotracer.

#### Results

NDL, NLL, H8 and PH1 were successfully synthesised and conjugated to NODASA, purified (> 95%) and characterised. Compounds were subsequently radiolabelled with gallium-68, optimal labelling conditions were explored to produce radiochemically pure products (> 99%) including suitable radiochemical yields and activity concentrations for future preclinical applications. All radiometal complexations demonstrated good integrity when challenged by 1000-times excess of EDTA (> 90%). [68Ga]Ga-PH1, [68Ga]Ga-NDL and [68Ga]Ga-NLL were stable for up to 2 h (> 90%) in human plasma whereas 68Ga-H8 degraded rapidly (< 40% remained intact after 30 min). All compounds showed minimal RBC interaction (< 10%) and plasma protein binding ([68Ga]Ga-NDL/-NLL < 14%; [68Ga]Ga-PH1/-H8 < 9%), thereby mimicking a favourable expected bioavailability.

#### Conclusion

Following successful radiolabelling and in vitro characterisation, [68Ga]Ga-NDL and [68Ga]Ga-PH1 are deemed promising candidate PET-radiotracers. Upcoming bacterial cell uptake assays and micro positron emission tomography/computed tomography imaging studies in mice will further assess the potential of these compounds as bacterial-specific ([68Ga]Ga-NDL), or even MTB-specific ([68Ga]Ga-PH1) radiotracers.

### Re-treatment of benign thyroid diseases with radioactive therapy

**P. Rivombo^1^, N. Nyakale^1^**

^1^ *Department of Nuclear Medicine, Dr George Mukhari Academic hospital, Sefako Makgatho Health Science University, Pretoria, South Africa*

#### Background

Benign thyroid diseases are the most common thyroid conditions seen at our department. These include Graces’ disease, diffuse toxic goiter, toxic or nontoxic goiter and solitary hyperfunctioning thyroid nodules. Treatment options are antithyroid treatment, radioactive iodine and total or subtotal thyroidectomy. Radioactive iodine therapy has been used to treat benign thyroid diseases for many years since the 1970s. Low dose of I-131 is used for treatment of benign thyroid diseases.

#### Aim

The aim of this study was to assess the effectiveness of I-131 therapy in benign thyroid diseases in our setting.

#### Methods

A retrospective analysis was carried out for patients with benign thyroid conditions who underwent single or double I-131 therapy for benign thyroid disease at our department between January 2009 and December 2021. The thyroid function status of the patients was also documented prior to discharge.

#### Results

A total of 127 patients were included in the study. The patients included in the study had the following conditions: Graves’ disease 81% (*n* = 104), toxic multinodular goitre 10% (*n* = 13), toxic adenoma 6% (*n* = 7), Marine Lenhard syndrome 2% (*n* = 3) and autonomous nodule 1% (*n* = 1). They were treated with an empirical dose of I131, ranging from 10 mCi to 40 mCi. Seventy-four per cent (*n* = 94) of the patients responded to the single RAI therapy (*n* = 33 euthyroid; *n* = 61 hypothyroid). The remaining 26% (*n* = 33) required retreatment as they remained hyperthyroid. Second RAI therapy was administered to 88% (*n* = 29) of patients while 12% (*n* = 4) of the patient defaulted treatment. The majority of retreatment patients had Graves’ disease 90% (*n* = 26), 10% (*n* = 3) had toxic multinodular goitre and 3% (*n* = 1) had toxic adenoma. On discharge 66% (*n* = 83) of the patients were hypothyroid and 31% (*n* = 40) were euthyroid. Three per cent (*n* = 4) of the patients defaulted treatment before hypothyroid/euthyroid state could be achieved.

#### Conclusion

Graves’ disease patients showed majority of treatment failure compared with other benign thyroid conditions. This is contrary to the commonly reported literature, which state that multinodular goiter is more resistant than Graves’ disease. Hypothyroid state was archived in more than half of patients.

### Persistent Sestamibi retention on dual phase parathyroid scintigraphy in Hashimoto’s thyrioditis: A case report

**A. Venter^1^, K. Purbhoo^1^, M. Vangu^1^**

^1^ *University of the Witwatersrand, Johannesburg, South Africa*

#### Introduction

Parathyroid imaging plays a critical role in preoperative management of parathyroid lesions.^1^ It localises hyperfunctioning parathyroid tissue and specifically parathyroid adenomas with a high sensitivity and specificity.^2^

Parathyroid lesion detection with dual-phase Technetium-99m (Tc- 99m) Sestamibi (MIBI) scintigraphy is dependent on the differential washout of the tracer from the thyroid parenchyma. In some cases, such as in autoimmune thyroid disease (AITD) or in the case of type-1 amiodarone induced thyrotoxicosis (AIT-1), scintigraphy may show persistent uptake of the thyroid parenchyma, with no or minimal washout of tracer on delayed imaging, posing a challenge for interpretation.^3^

#### Presenting case

A 53-year-old female known with Hashimoto’s thyroiditis (HT) was referred for new onset hyperparathyroidism. Ultrasound had features of HT, bilateral hypodense thyroid nodules and bilateral lower pole parathyroid glands. Dual-phase parathyroid scintigraphy showed diffuse uptake in the thyroid gland parenchyma, with persistent tracer retention on delayed imaging.

#### Method

A combination of dual-tracer – dual-phase imaging with single photon computed tomography/computed tomography (SPECT/CT) with Tc-99m MIBI and subtraction using Tc-99m pertechnetate was performed.

#### Outcome

Dual-phase parathyroid scintigraphy showed diffuse uptake with persistent tracer retention in the thyroid gland parenchyma on 3-h imaging. A bulky, elongated left thyroid lobe was identified and no ectopic focus of abnormal uptake were seen. Single photon computed tomography/computed tomography reconstructed images confirmed the findings.

Thyroid scintigraphy showed an enlarged gland with inhomogeneous tracer uptake. A bulky left inferior pole and an impression of ‘cold’ nodules in the left superior pole and right inferior pole were appreciated.

Based on the subtraction analysis, a diagnosis of parathyroid lesion in the inferior pole of the left and right lobes were considered and ultrasound correlation ± FNA was recommended for ‘cold’ nodule correlation.

#### Discussion

Literature has described focal thyroid disease, subacute thyroiditis, AITD such as Graves’ disease, hypertrophic form of HT, as well as AIT-1, where MIBI is not equally washed out on delayed imaging. Specifically, AITD is a significant factor for the retention of MIBI. Very few cases exist in the literature of Hashimoto’s thyroiditis as a cause of persistent MIBI retention.^3,4^

Theoretically, the above-mentioned factors increase false negative findings, as a result of reduced visibility of small parathyroid adenomas or parathyroid hyperplasia.^3^

#### Conclusion

This case demonstrated that AITD such as HT should be included in the list of differentials for persistent thyroid gland retention of MIBI, resulting in decreased sensitivity for the visualisation of hyperfunctioning parathyroid lesions on dual-phase parathyroid scintigraphy.

### Late presentation of congenital hyperinsulinaemia diagnosed on a 18-fluoro-L-3,4-dihydroxyphenylalanine positron emissions tomography scan: First case at Universitas Academic Hospital

**W. Endres^1^, T. Tebeila^1^, G. Engelbrecht^1^, O. Evbuomwan^1^**

^1^ *University of the Free State, Bloemfontein, South Africa*

#### Introduction

Congenital hyperinsulinism (CHI) is a clinical entity that presents as persistent hypoglycaemia in infants and children. Congenital hyperinsulinism classically presents at birth and poses a high risk for hypoglycaemic brain injury and neurodevelopmental abnormalities; thus, swift diagnosis and treatment are extremely important. Although the classical presentation is at birth, CHI covers a wide clinical spectrum, often leading to presentation after infancy.

Under normal physiological conditions, insulin secretion is typically suppressed if the blood glucose concentration is lower than 5.0 mmoL/L.

In patients with CHI, this normal physiological pathway is disrupted, resulting in inappropriate insulin secretion with a failure to suppress insulin secretion.

Diagnosis typically relies on a combination of clinical evaluation, laboratory investigations, molecular genetic testing and advanced imaging modalities such as 18-fluoro-L-3,4- dihydroxyphenylalanine positron emission tomography (18-F-DOPA) positron emission tomography/computed tomography (PET/CT) scan.

The role of 18-F-DOPA in CHI mainly centres on confirmation of diagnosis with or without molecular genetic testing and in distinguishing diffuse disease from a localised pancreatic disease. The latter plays an essential role in deciding the treatment path for the patient, as patients with focal disease benefit from surgical management and those with diffuse disease benefit from medical treatment with drugs like diazoxide.

We present our first case of CHI at Universitas academic hospital. An 8-month-old female patient was referred from Bongani regional hospital with a history of persistent hypoglycaemia and recurrent diffuse tonic-clonic seizures. This late presentation of CHI posed a diagnostic dilemma because, classically, patients with CHI will present around birth with symptoms. After initial laboratory and conventional radiological investigations were inconclusive, the patient was referred to the Department of Nuclear Medicine for F-18-DOPA PET/CT.

#### Method

We retrospectively reviewed the findings of the 18-F-DOPA PET/CT study and reviewed the images for the intensity and pattern of uptake.

#### Results

Positron emission tomography/computed tomography images of the abdomen and pelvis revealed higher than normal physiological uptake throughout the pancreas, with the head having the highest intensity. No other abnormal findings were noticed.

#### Conclusion

This case report shows the value of F-18-DOPA PET/CT in the confirmation of CHI. The diffuse nature in this particular case prompted the referral doctors to commence medical treatment and not surgical treatment. The patient was subsequently initiated on a trial of diazoxide and hydrochlorothiazide for 5 days. After showing an initial positive response, the child was discharged.

### Ga-68 dotatate avid ectopic adrenocorticotropic hormone secreting pulmonary carcinoid tumour detected on PET/CT

**L. Harry^1^**

^1^ *Inkosi Albert Luthuli Hospital, Durban, South Africa*

Tumours causing ectopic Cushing’s syndrome (ECS) are rare and cause excess cortisol as well as adrenocorticotropic hormone (ACTH) over-production with distinct clinical features. Pulmonary carcinoids tend to be more common than other causes of ectopic ACTH syndrome. Ectopic ACTH tumours are often not visible with conventional imaging, however depending on the histology, Gallium-68-DOTATATE, DOTATOC and DOTANOC positron emission tomography/computed tomography reportedly exhibits greater sensitivity in identifying an ectopic ACTH tumour source.

Here we present a 39 year male who presented with biochemical abnormalities and new onset diabetes and hypertension; a diagnostic work up for Cushing syndrome was undertaken; and it was found that 24-h urine free cortisol, midnight cortisol and ATCH levels were elevated. The MRI showed no pituitary lesion and CT was unremarkable. This alluded to an ACTH-dependent aetiology and the patient was referred for a [68Ga] -DOTATATE PET/CT scan to assist in the detection and/or localisation of a possible ACTH-dependent primary lesion. The scan showed pathological uptake in a parenchymal nodule in the right lower lobe, thought likely to represent the ACTH-secreting tumour. This was confirmed on histopathology post-surgical intervention. Patient had resolution of symptoms and was placed on surveillance. A repeat [68Ga] -DOTATATE PET/CT scan was performed 4 months later to assess for residual tumour, which revealed residual post-surgical inflammatory changes in the region of the right lobectomy.

Ectopic ACTH secretion from pulmonary carcinoid tumours (typical or atypical) can present as a challenge in diagnosis and management; however [68Ga]-DOTATATE can be useful as an initial diagnostic modality in this rare condition, which has a high mortality and morbidity.

### Does bone scintigraphy change clinical management in patients with lung cancer?

**T. Nxasana^1^, L. Harry^1^, L. Gabela^1^, B. Hadebe^1^, M. Patel^1^, V. Pillay^1^, S. Masikane^1^, M. Vorster^1^**

^1^ *University of KwaZulu-Natal, Durban, South Africa*

Lung cancer is the third most common cancer in South African men, with a lifetime risk of 1 in 98. According to the ESMO cancer guidelines, the standard work up includes computed tomography, X-rays, positron emission tomography/computed tomography (PET/CT) and bone scintigraphy.

Bone scintigraphy is commonly used in oncology for assessment of primary and secondary skeletal malignancies. In cancers that are classically characterised by osteoblastic skeletal metastases, bone scintigraphy is known to have high sensitivity for skeletal metastases. This is in contrast to other malignancies that typically have osteolytic bone metastasis or mixed osteolytic/osteoblastic skeletal lesions such as lung carcinoma. The diagnostic yield for these scans is lower on bone scintigraphy. The aim of this retrospective review is to evaluate the utility of bone scintigraphy in management of patients with lung carcinoma.

#### Materials and methods

A review was carried out from patient records at Inkosi Albert Luthuli central Hospital, nuclear medicine department from 2017 to 2022. The study was performed as retrospective analysis of patients with biopsy proven lung cancer who were referred for bone scan. All patients underwent bone scintigraphy in the nuclear medicine department at Inkosi Albert Luthuli Central Hospital. Image acquisition was performed on a Siemens biograph single photon emission computed tomography/computed tomography (SPECT/CT) camera. The computed tomography (CT) scan was unenhanced and CT parameters were 120 kev 50 mAs. The bone scan was acquired 2–4 h after an injected dose of 20 mci of 99mTc-Methylene Diphosphate. Regional SPECT/CT of the area of interest was acquired at 3 min/bed position.

#### Results

The study had 40 participants, 33 males and 7 females, with an age ranging from 45 to 83 years. Histology demonstrated non-small cell carcinoma for all of the participants, with the exception of two patients (who demonstrated adenocarcinoma). Our results demonstrated that bone scintigraphy influenced patient staging in 25% of cases and changed management in 17.5% of the cases. Serum ALP results were available in only 8/40 (20%) patients and was increased in all of these patients. (Detailed statistical analysis to follow).

#### Conclusion

In our study population, bone scintigraphy changed staging in 25% of patients with lung cancer and influenced management in 17.5%. Bone scintigraphy may not be the optimal nuclear medicine study and further studies are needed to compare the use of bone scintigraphy to that of 18F-FDG PET/CT in lung cancer patients.

### The case of multiple periarticular masses on bone scan

**S. Masikane^1^, L. Harry^1^, V. Pillay^1^, L. Gabela^1^, T. Nxasane^1^, B. Hadebe^1^, M. Patel^1^, M. Vorster^1^**

^1^ *UKZN -IALCH, Durban, South Africa*

Tumoural calcinosis is a rare disorder that was initially described as an arthropathy of calcium pyrophosphate dihydrate deposition. It tends to occur more predominantly in the periarticular location of the extensor aspects of the large joints. It is a benign familial metabolic dysfunction of phosphate regulation, resulting in high levels of phosphate in the plasma leading to calcific deposits of hydroxyapatite or amorphous calcium phosphate crystals into soft tissues. It can also result from other conditions such as renal failure, dialysis and hypervitaminosis D.

The index case is a patient operated in 2019 for tumoural calcinosis of the right knee, now referred to our department with painless periarticular swellings of the right femur and right elbow. Biochemically, the patient was found to have an elevated phosphate level, with normal calcium and parathyroid hormone (PTH) levels. Technetium-99m methylene diphosphate (99mTc MDP) bone scintigraphy was undertaken, confirming the findings of tumoural calcinosis in the suspected sites, as well as identifying other sites of involvement.

The use of bone scintigraphy has been proven to have a good sensitivity, and its ability to scan the whole body in one acquisition gives the modality the added advantage of identifying tumoural calcinosis skip lesions. This was well demonstrated with the above case where the patient was treated with multiple surgeries based on radiological findings, which were isolated to the area of complaint. Multiple skip lesions were identified by using bone scintigraphy (inclusive of bone marrow involvement), which resulted in change of the management approach in the patient.

Bone scintigraphy is therefore highly recommended, especially in patient with tumoural calcinosis, as it is able to identify lesions both in the suspected and other (skip lesion) sites.

### Evaluation of the added value of a diuretic renogram in the assessment of renal function prior to targeted radionuclide therapy

**L. Gabela^1^, L. Harry^1^, T. Nxasana^1^, M. Patel^1^, S. Masikane^1^, V. Pillay^1^, M. Vorster^1^**

^1^ *University of KwaZulu-Natal, Durban, South Africa*

#### Introduction and study aim

Lutetium-177 (Lu-177) is a radionuclide with unique theranostic properties that make it ideal for radionuclide therapy. The use of Lu-177 has shown promising results in the treatment of different types of cancers, including neuroendocrine tumours and prostate cancer.

In order for patients to qualify for Lutetium-177 radionuclide therapy a renogram to assess kidney function and rule out obstruction is required. Other tests including a Ga-68 prostate-specific membrane antigen (PSMA) is also performed.

This study aimed to compare the effectiveness of Ga-68 PSMA and Dotatate-derived split renal function with diuretic renogram-based split function in assessing the renal function.

A retrospective analysis (visual and quantitative) was conducted on 21 patients with histologically confirmed prostate cancer who were metastatic castration-resistant prostate cancer (mCRPC) and 10 patients with histologically confirmed well-differentiated NET- or moderately differentiated NETs, G1 or G2 and not amenable to curative surgery. Baseline renal function, renogram parameters and 68 Ga PSMA and Dotatate kidney findings were assessed. Renogram, Ga-68 PSMA and Ga -68 Dotatate were performed within a period of ± 1 week of each other. Diuretic renogram split function was calculated with symbia net software.

To calculate relative Ga-68 PSMA and Dotatate distribution in both kidneys, volumes of interest were drawn and generated using syngo.via software. The volume and the mean standard uptake value (SUVmean) were then used for calculating the split renal function (SRF).

Of the 31 patients with a mean age of 61 years two were noticed to have a difference in split function of more than 10% between the duiretic renogram and Ga-68 PSMA and Dotatate-derived split renal function. The balance of the patient’s demonstrated congruence between the diuretic renogram and Ga-68 PSMA and Dotatate-derived split renal function. Concordance was also noticed between renal abnormalities on diuretic renogram and Ga-68 PSMA and Dotatate scans.

The results showed that Ga-68 PSMA and Dotatate-derived split function had a high correlation with diuretic renogram-based split function in assessing renal function. Moreover, these tests also provided additional information regarding the functional status of the kidneys.

Apart from assessing function of the kidneys, anatomical defects can also be visually assessed.

Therefore, it is suggested that Ga-68 PSMA and Dotatate-derived split renal function can be used as an alternative to diuretic renogram-based split function.

### Irradiation of yttrium microspheres at the IRT-T reactor

**V. Sadkin^1^, E. Nesterov^1^, A. Naymushin^1^**

^1^ *Tomsk Polytechnic University, Tomsk, Russia*

National Research Tomsk Polytechnic University (TPU), together with an industrial partner (BEBIG), started producing yttrium microspheres at its research nuclear reactor this fall. This radiopharmaceutical is intended for the treatment of liver cancer in inoperable patients and, unlike analogues, it destroys the tumour in a targeted manner without affecting healthy organs and tissues. Irradiation of yttrium-89 microspheres will be carried out at the IRT-Treactor, power 6 MW, neutron flux 1.7 * 1014 neutron/cm^2^. To obtain one dose of the final preparation, a weighed portion of 0.1 g of yttrium microspheres is irradiated. Microspheres are processed using distilled water, alcohol and hydrochloric acid. Yttrium microspheres are injected into the patient’s bloodstream, which delivers them directly to the tumour. After delivery, the microspheres block the access of blood with oxygen to the metastases, in parallel acting on them with beta radiation. In the Russian Federation, this method of treatment is not yet massively applied.

The industrial production of yttrium microspheres began in the fall of 2020. There have already been test deliveries to Moscow clinics. The drug is registered and has permits for use in clinical practice. On 09 April 2021 at A. Tsyb Medical Radiological Research Center for the first time in Russia, clinical trials of the method of radioembolisation of tumours with domestic microspheres produced by the Russian company ‘Bebig’ began. Four operations were performed at once on patients with inoperable forms of liver cancer.

### Physiological biodistribution of Ga-68 DOTA-NOC in patients with neuroendocrine tumours: Influence of blood glucose level, somatostatin analogue therapy and imaging time

**G. Siwawa^1^, J. Warwick^2^, B. Berndorfler^3^**

^1^ *Nuclear Medicine Division, Department of Medical Imaging and Clinical Oncology, Stellenbosch University, Cape Town, South Africa*

^2^ *Nuclear Medicine Division, Department of Medical Imaging and Clinical Oncology, Stellenbosch University, Cape Town, South Africa*

^3^ *Nuclear Medicine Division, Department of Medical Imaging and Clinical Oncology, Stellenbosch University, Cape Town, South Africa*

#### Background

A common feature of neuroendocrine tumours (NETs) is that they express somatostatin receptors (SSRTs). Radiotracers such as Ga-68 DOTA-NOC, Ga-68 DOTA-TATE and Ga-68 DOTA- TOC are SSRT analogues and have proven very useful in imaging patients with NET.

The SSRTs are also found in a number of normal tissues. Differentiating pathological uptake from normal uptake in organs can be challenging when interpreting Ga-68 DOTA scans. Detailed reference ranges for the physiological uptake in organs has been published for Ga-68 DOTA-TATE and Ga-68 DOTA- TOC, but data are scarce for Ga-68 DOTA-NOC.

Somatostatin receptors receptor expression by normal organs demonstrates some intra-individual variation and may be affected by blood glucose levels, use of somatostatin analogue therapy or the delay in imaging time.

The aim of this study is to provide detailed reference ranges for the physiological uptake in organs for Ga-68 DOTA-NOC and compare these with limited prior literature. In addition, we will evaluate the effect of blood glucose, somatostatin analogue therapy and imaging time delay on normal Ga-68 DOTA-NOC uptake.

#### Methods

Patients having undergone Ga-68 DOTA-NOC positron emission tomography/computed tomography (PET/CT) scans at the NuMeRI Node for Infection Imaging PET/CT centre between 2019 and 2022 will be retrospectively selected. In patients with more than one Ga-68 DOTA-NOC scan only the first study will be included. The clinical history, blood glucose level, any recent somatostatin analogue therapy and the time delay between injection and imaging will be documented.

Uptake measurements will be captured by drawing 3D volumes of interest (VOI) in a number of organs. The maximum standard uptake value (SUVmax) for each organ will be documented, making sure that the VOI is drawn within the organ boundary and excludes any vascular structures. Organs with known tumour involvement will be excluded.

#### Results

We expect to provide detailed reference ranges for physiological organ uptake of Ga-68 DOTA-NOC. It is expected that certain values will be higher than what was previously documented in literature, for example for the uncinate process. We also expect that blood glucose levels, somatostatin analogue therapy and delay in imaging time to have a measurable effect on the physiological Ga-68 DOTA-NOC uptake in organs.

#### Conclusion

More comprehensive reference ranges for physiological organ uptake of Ga-68 DOTA-NOC and improving our understanding of the effects of blood glucose levels, somatostatin analogue therapy and delay in imaging time on normal organ uptake, we expect to enhance the interpretation of Ga-68 DOTA-NOC PET/CT studies.

### Needle in a haystack-malignant insulinoma

**P. Mokemane^1^, A. Ismail^1^, S. Padiachy^1^**

^1^ *Charlotte Maxeke Johannesburg Academic Hospital, Johannesburg, South Africa*

#### Introduction

An insulinoma is a neuroendocrine tumour localised in the pancreas that produces excessive amounts of insulin. They are usually benign and are a rare occurrence with approximately four cases per million. Malignant insulinomas are remarkably rare accounting for 10% of all cases. Nuclear medicine plays a useful role in the localisation of insulinomas using Gallium 68 Dotatate positron emission tomography/computerised tomography (PET/CT).

#### Case study

A 45-year-old female with a history of retroviral disease presented with recurrent hypoglycaemia. A computerised tomography (CT) scan of the abdomen showed liver lesions with no pancreatic lesions or insulinoma. She was then referred for a Gallium 68 Dotatate PET/CT in May 2020. The patient underwent a Whipple and hepatectomy. She had a follow-up Gallium 68 Dotatate PET/CT in April 2021.

#### Results

The initial Gallium 68 Dotatate PET/CT performed in May 2020 showed avid disease in the body of the pancreas as well as a large liver lesion. After undergoing surgery, a CT scan performed in March 2021 showed mesenteric lymph nodes and multiple liver lesions. This prompted a second Gallium 68 Dotatate PET/CT in April 2021 for restaging and recurrence. The scan showed extensive hepatic and skeletal disease as well as possible recurrence in the body and tail of the pancreas.

#### Conclusion

As a result of the size of insulinomas as well as the fact that they can occur in any part of the pancreas, they are often hard to detect. Gallium 68 Dotatate PET/CT scans are very useful in this regard because of their high affinity to somatostatin receptors. This makes it a viable option for insulinoma localisation within the pancreas and identification of other sites of metastases as demonstrated in this case study. Furthermore, it can improve diagnostic accuracy and surgical planning for patients with this rare and elusive tumour.

### Differences in tumour aggressiveness based on molecular subtype and race measured by [18F]FDG PET metabolic metrics in patients with invasive carcinoma of the breast

**S. Abubakar^1^, S. More^2^, N. Tag^3^, A. Olabinjo^4^, A. Isah^5^, L. Ismaheel^6^**

^1^ *SQCCCRC, Al-Khoud, Oman*

^2^ *UCT and GSH, Cape Town, South Africa*

^3^ *SQUH, Al Khoud, Oman*

^4^ *AFH, Al Khoud, Oman*

^5^ *National Hospital, Abuja, Nigeria*

^6^ *Emory University, Atlanta, Georgia, United States of America*

#### Aim

To assess 2-deoxy-2-[18F]Fluoro-D-glucose ([18F]FDG) positron emission tomography with computed tomography (PET/CT) semi-quantitative parameters in locally advanced invasive ductal carcinoma (IDC) of the breast and the differences in these parameters based on molecular subtype and race.

#### Methods

[18F]Fluoro-D-glucose PET/CT images of women with treatment-naïve locally advanced IDC of the breast were reviewed. Qualitative reading of the scans was performed to determine the presence of regional and distant metastases. Semi-quantitative reading of the scans was performed by generating volume of interest using iso-contouring and a fixed relative threshold of 40% and the maximum standardised uptake value-body weight (SUVmax), metabolic tumour volume (MTV), and total lesion glycolysis (TLG) of the primary breast lesion were obtained. The hormone receptor status, HER2 status and biodata including race were retrieved from their medical records. Statistical analysis for differences in the parameters based on race and molecular subtype was performed. We categorised the patients by their self-identified race as black, mixed ancestry or white.

#### Results

A total of 127 staging [18F]FDG PET/CT were included comprising 81 (63.8%) mixed ancestry, 40 (31.5%) black and 6 (4.7%) white patients. Mean age was 51.5 years (s.d. 12.78) and there was no significant difference in age between the various groups. In the IDC group as a whole, the primary tumour SUVmax, MTV and TLG as well as presence of distant metastases were significantly higher in black patients (*p* = 0.004, 0.04, 0.005, 0.002, respectively). The primary tumour SUVmax, MTV and TLG were significantly higher in the basal subgroup (*p* < 0.001 in all). The primary tumour SUVmax and the presence of distant metastases were significantly higher in black patients in the luminal subgroup (*p* = 0.012 and 0.006, respectively), the SUVmax and TLG were significantly higher in black patients in the basal subgroup (*p* = 0.057 and 0.007, respectively).

#### Conclusion

Significant differences in [18F]FDG PET/CT parameters were seen in molecular subtype of IDC corresponding to known aggressiveness. The significantly higher parameters in black patients in IDC as a whole and in luminal and basal subtypes are suggestive and supportive of a more aggressive disease.

### Milk scan – Does longer scanning time increase the detection of reflux?

**F. Smith^1^, K. Hlongwa^2^**

^1^ *Red Cross War Memorial Children’s Hospital, Cape Town, South Africa*

^2^ *Red Cross War Memorial Children’s Hospital, Cape Town, South Africa*

#### Objective

The milk scan is a well-established technique for detecting gastro-oesophageal reflux (GER) and pulmonary aspiration. There is no universally accepted protocol for this study. At RXH, the dynamic imaging protocol for milk scans in search for GER has been to image for 30 min at 5 s/frame. However, internationally, imaging is acquired over 60 min at 5 s/frame. This bodes the question of whether we may have missed refluxes by not scanning for a further 30 min, thereby reducing the sensitivity of our protocol.

#### Method

We retrospectively reviewed 200 patients who presented for milk scans over a 13-month period from 01 November 2021 to 30 November 2022. A total of 100 patients were imaged with the 60-min protocol and 100 patients were imaged with the 30-min protocol. We compared the number of positive scan results in the 60-min protocol to the positive ones in the 30-min protocol. Then, we further analysed how many of the positive scans in the 60-min protocol showed reflux in the last 30 min of the scan. Has the 30-min protocol significantly reduced the sensitivity of the reflux search?

#### Results

In the patients scanned with the 60-min protocol, 65/100 patients had positive scans in comparison to 70/100 patients imaged with the 30-min protocol in the 13-month review period. In the patients who demonstrated positive studies in the 60-min protocol, 7/65 patients demonstrated reflux in the last 30 min of the study and 21/65 patients demonstrated reflux in the first 30 min and did not demonstrate reflux in the last 30 min, 37/65 patients demonstrated reflux throughout the 60-min study. In this review period of 13 months, there was no increase in the incidence of detecting reflux in our milk scans over the 60-min period in comparison to the 30-min period.

#### Conclusion

Increasing the milk scan protocol to 60 min did not demonstrate an increase in the incidence of detection of reflux in our centre. The significance of patients in whom reflux was detected in the last 30 min of the 60-min study needs to be reviewed in further studies.

### Lutetium-177 peptide receptor radionuclide therapy of de-differentiated thyroid cancer

**X. Khuzwayo^1^, N. Mlaba^1^, A. Gutta^1^, N. Nyakale^1^**

^1^ *Sefako Makgatho University of Health Sciences, South Africa*

#### Introduction

Well-differentiated thyroid cancer when originates from thyroid follicular epithelium and retains its biological characteristics. This allows for it to be treated with radioactive iodine therapy (RAI). About 5% of patients develop metastatic thyroid disease, which is refractory to RAI. Lutetium-177-labelled peptide receptor radionuclide therapy (PRRT) was explored as an alternative option.

#### Methods

Sixty-three-year-old female with follicular thyroid cancer post-thyroidectomy more than 10 years ago, presented with right hip pain because of a surgically irresectable mass in the right hemi-pelvis with lytic destruction of the right hip involving the lesser trochanter, acetabulum, ilium and pubic rami.

A diagnostic iodine-123 (I-123) whole-body scan (WBS) revealed uptake in the thyroid bed (thyroid remnant tissue), focal uptake in the left cervical lymph node and heterogenous uptake in the right hemi-pelvis, most intense over the right hip lesser trochanter. She received two cycles of I-131 therapy in 2019 at 150 mCi and 2021 at 200 mCi. Post 2nd cycle of I-131 therapy, the post-therapy no longer showed uptake in the right pelvic mass with exception of a focus in the lesser trochanter. A de-differentiated thyroid carcinoma was suspected.

F18-Fluoro-D-glucose (F18-FDG) positron emission tomography/computed tomography (PET/CT) scan revealed a destructive, locally infiltrative lesion in the right hemipelvis with abnormal, intense and homogenous uptake. A Ga-68-DOTATATE PET/CT scan demonstrated somatostatin receptor expression at a Krennig score 4 in the mass, congruent to the FDG uptake, with a mildly avid right inguinal lymph node. Post-workup for PRRT, she received a total of cycles of Lu-177-DOTATATE therapy.

#### Results

Post three cycles, her ECOG score improved from 3 to 1 and her post-therapy scans showed a reduction in the size and uptake of the pelvic mass. The post-therapy scan following her 4th cycle revealed stable disease. The Ga-68 DOTATATE was repeated as per protocol, at 6 months after her fourth cycle. This scan revealed progression of the lesion in right hemipelvis with regard to intensity and size.

#### Discussion and conclusion

Treatment options for de-differentiated thyroid cancer refractory to I-131 are limited and tend to show a suboptimal response. Peptide receptor radionuclide therapy with beta-emitting and alpha- emitting radionuclides serves as an alternative option in thyroid cancer lesions expressing somatostatin receptors. The relatively short range of Lu-177 may be a factor which results in a slow response given the size of the lesion of the above patient, and tandem treatment with Y-90 PRRT may need to be considered.

### Potentiometric studies of 117MSN-poly-HEDP and 177LU-poly-HEDP: In attempts to develop effective radiopharmaceuticals for bone metastases

**L. Sepini^1^, J. Zeevaart^1^, D. Jansen^1^, T. Ranqhai^2^, N. Jarvis^3^**

^1^ *South African Nuclear Energy Corporation, Pretoria, South Africa*

^2^ *University of Johannesburg, Auckland Park, South Africa*

^3^ *International Atomic Energy Agency, Vienna, Austria*

Secondary cancer tumour formation, often called metastases, remains one of the great scientific challenges in public health. Therapeutic radiopharmaceuticals for bone pain palliation play an important role in providing quality of life for cancer patients with metastatic bone cancer. They comprise a bone seeking bisphosphonate ligand and a radionuclide. The structural variations of the bisphosphonate affect effectiveness of the radiopharmaceutical to a great extent with the greatest shortfall being bioavailabity. A polymer ligand poly-HEDP was synthesised from its free acid form in relatively low yields. This study seeks to understand the in vivo chemistry of the [117mSn]Sn(II), [117mSn]Sn(IV), and [177Lu]Lu(III) complexed with poly-HEDP in blood plasma. The formation constants of Sn(II), Sn(IV) and Lu(III) were measured by glass electrode potentiometry at 25°C and *I* = 150 mM. This made possible the construction of a blood plasma model of poly-HEDP, determined with the aid of thermodynamic blood plasma modelling simulations. The Sn(IV)-poly-HEDP complex was shown to be unstable, with a 100% dissociation, while Sn(II)-poly-HEDP showed much improved stability with 10% of the metal ion remaining bound to the ligand. However, 97% of the Lu(III) remained complexed to the ligand with less competition from the physiological ligands in vivo. The synthesis of poly-HEDP from poly acrylic acid was achieved successfully. A thermodynamic blood plasma simulation revealed that Lu(III) is practically quantitatively complexed to poly-HEDP as compared with Sn(II) and Sn(IV) in vivo.

### FDG-PET/CT imaging of tuberculosis in children

**D. Smith-Tsibolane^1,2^, C. Burger^1,2^, C. Le Roux^1,2^, A. Doruyter^1,2^, P. Goussard^1,2^**

^1^ *Stellenbosch University, Somerset West, South Africa*

^2^ *Tygerberg Hospital, Cape Town, South Africa*

#### Background

Tuberculosis (TB) causes significant morbidity and is a leading cause of death globally. Young children are at higher risk of severe forms of TB, including disseminated disease and TB meningitis. Up to a fifth of patients with TB in high-incidence countries are younger than 16 years, with a high mortality in untreated children. The diagnosis of childhood TB is challenging as the disease is often paucibacillary, and sampling of specimens is difficult, causing poor sensitivity of available pathogen-based tests. Tuberculosis can mimic numerous other pathologies, complicating differentiation between these, active TB or latent disease. The diagnosis of active TB is crucial to initiate adequate treatment timeously, avoid complications and prevent transmission. Evaluation of CXR has several limitations; therefore, cross-sectional imaging, e.g. CT, should be considered in the diagnostic evaluation of a symptomatic child. Positron emission tomography combined with (X-ray) computed tomography (PET/CT) using fluorine-18 fluorodeoxyglucose (FDG) has been shown to detect active TB with accuracy equal or superior to other conventional imaging modalities and its role in complicated TB is expanding. Fluorodeoxyglucose PET/CT can effectively identify sites of intrathoracic and extrathoracic TB, assess disease activity, assist in differentiating between active and latent disease, monitor response to therapy, and identify potential biopsy targets. There is a paucity of literature on PET/CT in childhood TB. The efficacy of FDG PET/CT in the paediatric population will be the focus of this case series.

#### Methods

A retrospective case series of children between the ages of 3 months and 16 years who presented at Tygerberg Hospital between January 2000 and August 2020 and who had a FDG PET/CT scan for suspected tuberculosis will be performed. The PET/CT scans will be reviewed by a qualified nuclear physician and compared with the findings of the CT scan (if available) and chest X-ray (CXR). Clinical data will be collated from patient folders and bronchoscopy and laboratory databases. The data will be analysed using standard descriptive and non-parametric statistical techniques.

#### Results

Fluorodeoxyglucose PET/CT was used mainly for four indications: to assist in the diagnosis of MDR TB, identify suitable biopsy sites, evaluate for ongoing disease in complicated TB, and for disseminated disease. The demographic profile and PET/CT findings (including their impact on management) will be presented.

#### Conclusion

Fluorodeoxyglucose PET/CT is expected to impact the management of selected paediatric tuberculosis cases by demonstrating extent and activity of disease, identifying extrathoracic sites of disease and guiding biopsies.

### The role of [18F]FDG-PET/CT imaging in selecting candidates for radionuclide therapies targeting PSMA

**B. Hadebe-Chonco^1^, M. Vorster^1^, L. Harry^1^, L. Gabela^1^, M. Patel^1^, V. Pillay^1^, S. Masikane^1^, T. Nxasana^1^**

^1^ *Inkosi Albert Luthuli Central Hospital, Umkhumban, South Africa*

^2^ *University of KwaZulu-Natal, Durban, South Africa*

#### Background

Tumour sites with low prostate-specific membrane antigen (PSMA)-avidity and high fluorodeoxyglucose (FDG)-avidity represent sites of aggressive disease, which cannot be effectively targeted by radioligand therapy. The presence of discordant lesions seen in very advanced mCRPC has a poorer prognosis. The VISION and TheraP trials where patients were required to have highly PSMA avid disease with no discordant lesions on [18F]FDG-PET/CT (positron emission tomography/computed tomography) prior to [177Lu]- PSMA therapy showed higher response rates. Ideally, all patients who are potential candidates for PSMA targeted radionuclide therapies should undergo [18F]FDG PET/CT together with [68Ga] PSMA PET/CT to detect sites of low PSMA-avidity and high FDG-avidity, which preclude the use of targeted by radioligand therapy; however, this is not always possible because of the high cost involved. The aim of this study was to assess the impact of [18F]FDG-PET/CT in selecting patients eligible for PSMA targeted radionuclide therapy.

#### Methods

Twenty patients with metastatic castration resistant prostate referred for PSMA radioligand therapy underwent [18F]FDG-PET and [68Ga] PSMA-PET with low-dose CT to assess if they were candidates for PSMA radioligand therapy. The studies were performed within 2 months of each other. Images were retrospectively analysed using MIM encore to compare differences in tumour bulk volume (TBV) between the [18F]FDG-PET and [68Ga] PSMA-PET/CT, which were then correlated with Gleason score, PSA level and survival.

#### Results

There were 20 men with cancer 44–74 (mean age 66 ± 8) with adenocarcinoma of the prostate were included, the average Gleason score was 8 (range 7–10). All patients had pelvic lymph node metastasis and widespread skeletal disease involvement on [68Ga] PSMA-PET. In 19/20 patients, no mismatch findings could be identified. One (1) patient (4.5%) with a Gleason score of 10 had FDG+/PSMA− lesions in abdominal lymph nodes, which precluded them from [177Lu]-PSMA therapy. This cost R 500 000 worth of [18F]FDG-PET/CT (20 scans) to save R 240 000 worth of [177Lu]-PSMA RLT – assuming that the patient would have received four cycles.

#### Conclusions

Our analysis showed that FDG+/PSMA−lesions occurred in 5% of patients prior to RLT, and this was in a patient with Gleason score of 10. Therefore, the cost of FDG PET/CT may be justified in patients with high Gleason scores. However, there is evidence that RLT still prolongs survival even in patients with low PSMA avidity as compared to best standard of care. Future studies need to look at the cost-effectiveness of RLT in patients with discordant-FDG positive disease with no other therapeutic options.

### Promoting a validation, protocol of radio-HPLC methods for the determination of radiochemical purity for small size gallium-68 labelled peptide derivatives

**B. Marjanovic-Painter^1^, J. Kleynhans^2^, J. Suthiram^1^, J. Zeevaart^1,3^, E. Rohwer^4^, T. Ebenhan^1,3,5^**

^1^ *Necsa, Pelindaba, South Africa*

^2^ *Radiopharmaceutical Research, Catholic University of Leuven, Leuven, Belgium*

^3^ *Nuclear Medicine Research Infrastructure NPC, Pretoria, South Africa*

^4^ *Department of Chemistry, University of Pretoria, Pretoria, South Africa*

^5^ *Department of Nuclear Medicine, University of Pretoria, Pretoria, South Africa*

#### Introduction

Radiochemical purity is a test performed during the quality control of radiopharmaceuticals to quantify the content of radioactive impurities introduced or formed during labelling. Possible impurities and labelled products are separated using the high-performance liquid chromatography (HPLC) principle whereafter the radioactive nature of the sample can be detected using radio detectors. Very often because of this radioactive nature, non-active reference standards, if available, are used to establish and validate HPLC separation method which, in most aspects, can be translated to analyses of radioactive components. However, the radioactive properties of these radiopharmaceuticals will require some specific tests or an adjusted setting concerning the validation of some parameters. In this work, we present step by step how validation parameters are investigated (accuracy, linearity, precision, robustness, stability of solutions) applicable to general radio-HPLC analytical methods, on an example of an already approved radiolabelled compound.

#### Methodology

This method is translatable to the determination of radiochemical purity for most small size gallium-68 labelled peptides or peptidomimetics. Validation parameters described are in accordance with European Pharmacopoeia and International Conference on Harmonisation (ICH) prescribed acceptance limits. High-performance liquid chromatography method was validated and provides evidence on accuracy, repeatability, intermediate precision, suitable system parameters, specificity and robustness. The limits of detection and quantification for the method are also provided.

#### Results

Validation parameters met all criteria as follows: Accuracy 100% ± 5, repeatability relative standard deviations (RSD) < 2.00%; intermediate precision RSD < 5.00%; theoretical plates *N* > 1000, capacity factor *k* > 2.00 specificity/resolution Rs > 1.50, symmetry As = 0.8–1.5. Robustness was established for column temperature (± 2 degrees Celsius), °C flow rate (± 10%) and mobile phase TFA (± 10%). Solution stability was established (short-term integrity and forced degradation/thermal decomposition).

#### Conclusion

A successful validation of radio-HPLC procedure suitable for conjugated peptides to allow gallium-68 radiolabelling is presented. Radiochemical purities > 95% can be trustfully measured. Although such procedures are often neglected during research, good manufacturing practices and the necessary validations of analytical equipment are non-negotiable when radiopharmaceuticals are prepared for administration to patients.

### The development of lyophilised kit preparations in a research setting: Challenges and lessons learnt

**J. Suthiram^1^, B. Marjanovic-Painter^1^, T. Ebenhan^2,3^, J. Wagener^1^, J. Zeevaart^1,2,4^**

^1^ *Applied Radiation, The South African Nuclear Energy Corporation (Necsa), Pretoria, South Africa*

^2^ *Nuclear Medicine Research Infrastructure (NuMeRI), Steve Biko Academic Hospital, Pretoria, South Africa*

^3^ *Department of Nuclear Medicine, University of Pretoria, Pretoria, South Africa*

^4^ *Preclinical Drug Development Platform, Department of Science and Technology, North-West University, Potchefstroom, South Africa*

In recent years, there has been a considerable increase in investigations towards the development of lyophilised (cold) kit formulations as an alternative to the automated synthesis of 68Ga radiopharmaceuticals. Kit-derived radiopharmaceutical preparation is not only user-friendly, robust and cost-effective but also offers the convenience of in-house preparation using generator produced radioactivity. It is these advantages that have contributed to the extensive use and success of 99mTc based single photon emission computed tomography (SPECT)-radiopharmaceuticals. As this approach involves efficient radiolabelling techniques and minimal equipment it is now considered to ease the more technical, manual radiosynthesis of 68Ga-based positron emission tomography (PET)-radiopharmaceuticals. To date, commercially available kit-based 68Ga-radiopharmaceuticals coupled with market authorised 68Ge/68Ga-generator systems have already demonstrated feasibility. Cold kits consist of freeze dried non-radioactive substrates and reagents (biological moiety, chelator-, buffer-, stabiliser- and bulking agent). The gold standard follows a ‘shake-and-shoot’ preparation that facilitates one-step ‘instant’ radiolabelling. Producing lyophilised kits of acceptable quality requires consideration of all aspects of the design and manufacturing process. Process validation evaluates data gathered over the product’s lifecycle to confirm that the process reliably generates a product to a determined standard. Sterile filtration, aseptic dispensing and freeze-drying are the main steps validated in kit production.

Herein, we report our experiences in the process validation of the GMP-manufacture of a lyophilised kit for PET imaging. Several kit batches were produced over a few months (different operators) and analysed using the validated quality control methods for visual inspection, pH (cold/radiolabelled), loss on drying, mass of pellet, concentration and radiochemical purity/yield. Filters were consistently integral and did not impact the concentration or kit pH (solution acidity). Batches subjected to sterility assessment were found to have no bacterial growth. Following visual inspection, the average yield of kits was approximately 80% with minor, major and critical defect numbers within acceptable limits. The laboratory scale, benchtop freeze dryer used in this study’s setting did not provide a repeatable and robust production method. The evaluation revealed the impact of limited freeze dryer functionality whereby shelf temperature cannot be controlled to facilitate pre-freezing and only minimal adjustments to temperature and vacuum pressure settings are possible. This finding is of particular importance for buffer solutions with complex freeze-drying properties.

These factors together with the duration of pre-freezing and freeze-drying had a noteworthy influence on the visual appearance of the kit pellet and the kit pellet moisture – these results are highly relevant whenever novel PET-radiopharmaceuticals are under consideration for translation to routine manufacture.

### The added advantage of 18-flouro deoxy glucose, positron emission tomography/computed tomography in a solitory bone scan lesion in breast cancer

**P. Nemutaduni^1^, N. Nyakale^2^, N. Mlaba^3^**

^1^ *SMU, Pretoria, South Africa*

^2^ *SMU, Pretoria, South Africa*

^3^ *SMU, Pretoria, South Africa*

The article exhibits the added benefit of 18-flourodeoxyglucose, positron emission tomography imaging coupled with computed tomography in a patient with a solitary lesion in the spine on initial staging with single photon emission computed tomography/computed tomography (SPECT/CT) imaging.

#### Background

Bone scan remains the standard of care for breast cancer patient staging. Positron emission tomography together with computed tomography (PET/CT) is extensively used as an imaging tool for staging, re-staging, and treatment response and evaluating of recurrent disease. Breast cancer is characterised into histological sub groups, which exists at molecular level based on oestrogen and progesterone gene heterogeneity. One of the sub types is luminal B, which encompass 15% – 20% of breast malignancy. Luminal-B tumours are mostly characterised by highly histological grade, aggressive, high proliferative index and associated with worse prognosis. Hann N et al. found that on a lesion basis, whole-body fluro-deoxyglucose (FDG) PET/CT was more sensitive and equally specific for detection of bone metastases when compared with bone scintigraphy.

#### Material and methods

A case of a 69-year-old female patient with histologically proven luminal B, stage III A left breast cancer, ki-67 of more than 14%. The patient had Tc-99m methylene diphosphate (MDP) bone scan and was imaged using whole-body scintigraphy, which was subsequently followed by FDG PET/CT.

#### Results

Methylene diphosphate whole-body bone scan imaging showed an isolated intense uptake in the spinous lesion at the level of T1, which was localised in the posterior elements; however, because of high intense uptake coupled with relative hypo density on CT, right axillary lymph nodes and high clinical staging; further investigation with 18F FDG PET/CT was performed. History of trauma was excluded. Findings on PET/CT showed widespread metastases involving the skeletal and further metastases in the lymphatic and visceral organs.

#### Conclusion

Results demonstrate that in a patient with highly clinical and Scintigraphic suspicious lesions, a negative bone scan cannot conclusively exclude metastases. Additional imaging with FDG PET/CT is an essential advantage for adequate management of the patient.

### Value of the health assessment questionnaire disability index score in evaluation of radiosynoviorthesis treatment response in patients with refractory synovitis

**N. Jacobs^1^, O. Kolade^1^, B. Hodkinson^2^, S. More^1^**

^1^ *Division of Nuclear Medicine, University of Cape Town, Cape Town, South Africa*

^2^ *Division of Rheumatology, University of Cape Town, Cape Town, South Africa*

#### Background

Radiosynoviorthesis (RSO) is a medical technique, which utilises the administration of intra-articular radiopharmaceuticals to improve the effects of synovitis in inflammatory and haematological joint diseases. Response to RSO is routinely assessed with a Visual Analogue Scale (VAS). A 50% or more reduction in the pre-treatment VAS value 6 weeks post-therapy is considered successful. The Health Assessment Questionnaire (HAQ) score is a tool validated in patients with rheumatological diseases and haemophiliac arthropathy to assess treatment response to conventional medical therapy. There is limited evidence for the use of the HAQ score to assess response after RSO.

#### Aim

To determine the accuracy of the HAQ score in the response assessment of patients treated with radiosynoviorthesis for refractory synovitis.

#### Method

A retrospective study of patients with refractory synovitis because of inflammatory or haematological joint disease was conducted. They received radiosynoviorthesis treatment at Groote Schuur Hospital between 2017 and 2023. Active synovitis was confirmed through radiological, sonographic and/or scintigraphic imaging. Patients were clinically examined and were required to complete HAQ and VAS questionnaires prior to and 6 weeks after treatment. Patients without both sets of HAQ and VAS scores were excluded from the study.

#### Results

Of the 33 patients treated and 45 total treatments performed, 21 patients were included in the study, with a combined total of 24 treatments. Most patients were excluded because of incomplete HAQ and/or VAS scores. More than half of the patients suffered from rheumatoid arthritis (54%). Haemophilia was the second most common disease (25%). The remainder comprised patients with psoriatic arthritis, reactive arthritis and juvenile idiopathic reactive arthritis (20%). There was a 63% decrease in VAS scores post-RSO of 50% or more compared with the pre-treatment scores. A 25% improvement in the HAQ scores post-RSO treatment was identified. No significant correlation was found between the percentage change in VAS and HAQ scores in patients with successful RSO response.

#### Conclusion

There was a significant improvement in the HAQ score of patients receiving radiosynoviorthesis. However, the HAQ score alone cannot be used as an independent assessment tool in the evaluation of treatment response. Integration of the HAQ score with other assessment parameters, including relevant clinical and imaging criteria, is therefore required for accurate assessment of response to RSO.

### Radiopharmaceutical inventory management at the Nuclear Medicine Department of Dr. George Mukhari Academic Hospital

**T. Chidziwa^1^, M. Lundie^1^, B. Summers^1^**

^1^ *Sefako Makgatho Health Science University, Garankuwa, South Africa*

#### Background and objectives

Radiopharmaceutical products and procedures are highly expensive; hence, there is a need to reduce unnecessary costs incurred. An appropriate inventory management system is essential in ensuring traceability of each radiopharmaceutical product, prevention of stock outs and expired products. In this study, the inventory management system in place before the appointment of a radiopharmacist at the single photon emission computed tomography (SPECT) Nuclear Medicine Department (DNM) of Dr. George Mukhari Academic Hospital (DGMAH) was reviewed. An appropriate inventory management system was recommended, and SOPs and stock card systems were developed.

#### Methods

The study was descriptive and quantitative. In Phase 1, a review of the inventory system utilised in the department was conducted, baseline findings were examined according to GPP guidelines, and problem areas were detected. In Phase 2, the most appropriate reorder quantity, average daily consumption and lead times for each radiopharmaceutical product were calculated.

#### Results

The overall compliance per focus area at baseline (Phase 1) was as follows: inventory management (0%), availability of SOPs (16.7%), record keeping (33.3%), temperature monitoring (33.3%), physical storage (66.7%) and radiopharmacy security (66.7%). In Phase 2, the identified non-compliance indicators were rectified and recommendations were made.

#### Conclusion

The study revealed that the inventory management system at the DNM was informal. It was characterised by stock outs, expired medication, a lack of a stock card system, a lack of inventory management SOPs and ineffective record-keeping procedures. Interventions were introduced to address these gaps. Thus, an independent demand inventory system was employed by developing stock cards for reordering and appropriate record keeping allowing forecasting of stock requirements. A radiopharmacist was appointed as part of the research recommendations.

### The potential danger of a perfusion only SPECT CT study in the diagnosis of pulmonary embolism since the advent of COVID-19 infection

**O. Evbuomwan^1^, W. Endres^1^, T. Tebeila^1^, G. Engelbrecht^1^**

^1^ *University of the Free State, Bloemfontein, South Africa*

#### Introduction

In recent years, nuclear medicine imaging of pulmonary embolism has evolved, with VQ single photon computed tomography/computed tomography (SPECT/CT) being the latest technique. When for any reason the ventilation part of the study cannot be performed, the perfusion study is read along with the computed tomography (CT). It is well known from published studies in the literature that a perfusion only study is associated with a significantly lower diagnostic accuracy. In the initial period of the COVID-19 pandemic, a lot of nuclear medicine centres omitted the ventilation part of the study. The COVID-19 has been known to be associated with a lot of perfusion abnormalities, some with corresponding mosaic hypoattenuation on the CT, including pulmonary embolism. Some of these abnormalities reported with a CT only can mimic pulmonary embolism and further reduce the diagnostic accuracy of a perfusion SPECT/CT study. In this study, we looked at the data of known COVID-19 patients who had perfusion SPECT/CT studies and were erroneously diagnosed as having pulmonary embolism initially. We also looked at a small cohort of patients who performed perfusion only SPECT/CT during a period where we could not perform ventilation studies and compared their study with a repeat study performed with a ventilation.

#### Methods

This was a retrospective study where we reviewed the perfusion only SPECT/CT studies of nine recovered COVID-19 patients and four patients with unknown post-COVID-19 status. We compared these findings with a repeat study, which included ventilation studies.

#### Results

Thirteen cases were studied in total. All 13 cases had perfusion only SPECT/CT studies reported as being positive for pulmonary embolism. A review of their repeat studies with ventilation revealed that these defects were matched, and these matched defects were associated with mosaic hypo attenuation on CT. Some of these patients also had these matched defects persisting for over a year.

#### Conclusion

The COVID-19 pandemic might contribute to a further reduction in the diagnostic accuracy of a perfusion only SPECT/CT study. Our hypothesis is that perfusion only studies with a defect that corresponds to an area of subtle mosaic hypoattenuation should be at best reported as a non-diagnostic study, with appropriate follow-up management. We would, however, need bigger and well-organised prospective studies to validate this.

### 99mTc ethylenedicysteine-deoxyglucose (glucosamine) in the identification of active disease in patients with rheumatoid arthritis: A single-centre prospective study

**O. Evbuomwan^1^, J. Horn-Lodewyk^2^, G. Engelbrecht^1^, C. Driver^3^, B. Van Rensburg^1^, M. Labuschagne^1^**

^1^ *University of the Free State, Bloemfontein, South Africa*

^2^ *Central University of Technology, Bloemfontein, South Africa*

^3^ *Nuclear Energy Corporation South Africa, Pretoria, South Africa*

#### Introduction

Rheumatoid arthritis is a chronic inflammatory disease, which, if not treated properly, leads to irreversible joint damage, deformities and premature mortality. There are several investigations that can be used to detect and assess disease activity in patients with this condition. These include, clinical assessment, laboratory investigations and imaging. However, these different techniques have various shortcomings, and ideally a modality with a very high diagnostic accuracy is needed to detect disease activity, even at a subclinical level. The aim of this investigation was to evaluate the utility of 99mTc-labelled glucosamine (ethylenedicysteine-deoxyglucose) for the identification of active disease in patients with rheumatoid arthritis. The hypothesis is that this radiotracer could be a cost-effective, highly sensitive tool in the assessment of patients with rheumatoid arthritis.

#### Methods

This was a prospective study in which 22 participants with diagnosed active rheumatoid arthritis using the American College of Rheumatology/European League Against Rheumatism (ACR/EULAR) were recruited. Glucosamine ligand was synthesised according to standardised existing protocols in the literature described by Yang et al. Each participant was injected with 20 mCi–25 mCi of 99mTc-labelled glucosamine. Flow, blood pool and single photon emission computed tomography/computed tomography (SPECT/CT) imaging of the most symptomatic joints were performed, in addition to a delayed whole-body imaging 2 h after radiotracer injection. Joints were qualitatively assessed for abnormal increased uptake of the radiotracer. Degree of uptake was interpreted as normal, mild, moderate or severe using a 4-point scoring system.

#### Results

Twenty-two participants were recruited for the study. The median (IQR) age was 59 (49–68) years, and majority (95.5%) were females. All 22 participants had abnormal increased uptake of tracer in sites of known disease, including unknown sites. The SPECT/CT imaging localised tracer uptake specifically to the synovial space. Increased flow studies were associated with either Grade 3 or 2 disease on the delayed static images.

#### Conclusion

99mTc-labelled glucosamine was able to clearly detect active disease (synovitis) in all the known symptomatic joints of our patient population. The intensity of tracer uptake seems to be related to disease severity. It is yet to be known if this can also provide prognostic information.

### SPECT/CT whole-body bone scan time optimisation using image quality: A phantom study

**T. Mkhize^1,2^, M. Seabi^1^, S. Zwane^1^**

^1^ *Inkosi Albert Luthuli Central Hospital, Durban, South Africa*

^2^ *University of KwaZulu-Natal, Ethekwini, South Africa*

**Introduction:**

#### Aim

This study aimed to investigate the effect of different reconstruction protocols on image quality, while using varying scan times on single-photon emission computed tomography/computed tomography (SPECT/CT). The main goal was to establish the shortest possible acquisition time for clinical whole-body SPECT/CT while maintaining an acceptable image quality.

#### Methods

The National Electrical Manufacturers Association (NEMA) image quality phantom from PTW with five fillable spheres (diameters: 10 mm, 13 mm, 17 mm, 22 mm and 28 mm [37 mm sphere was unusable]) without the lung insert was used. The Siemens dual-head Intevo 6 gamma camera with a low energy-high resolution (LEHR) collimator was used for the Technitium-99 m energy. The spheres were filled with activity making a sphere-to-background concentration ratio of 8:1. Acquisition times were varied with a time per projection (TPP) of 5, 8 and the department’s 15 s (60 projections per head). These TPP’s gave a total of 1 bed acquisition of 5, 8 and 15 min. Images were reconstructed on a MIMS software workstation using the ordered subset expectation maximisation algorithm (OSEM), with 8 subsets and 4, 8 and 16 iterations with a 3D Gaussian filter smoothing of 0 mm–12 mm at full width half maximum (FWHM). The image quality components that were evaluated were contrast recovery (CR), signal-to-noise ratio (SNR) and background variability (BV).

#### Results

It was observed that the image quality statistics improved with increasing acquisition time. In respect of iterations, it was observed that the convergence of CR was observed at 8 iterations, while the higher 16 iterations tended to increase the reconstruction times significantly. The 3D Gaussian filter used for smoothing was found to be decreasing the noise at 12 mm FWHM. The BV improved with increased filter smoothing for all sphere sizes with noise decreasing as acquisition time increased. At a time of 8 s, the possibility of detecting the smallest sphere from the images was greater than 5 as per the rose score criterion.

#### Conclusion

This study was aimed at finding the optimal imaging time, which can lower/reduce the department’s scan times. From this study, we observed that the 8 s (8 min per bed) image qualities were similar with our departments 15 s (15 min per bed) imaging. Our protocol of 3 bed whole-body SPECT/CT total time of 45 min could be lowered by up to 21 min to a total whole-body scan time of 24 min. The optimal reconstruction parameter chosen from the results was 8 subsets, 8 iterations and 12 mm filter.

### Validation of planar gated bloodpool left ventricular ejection fraction processing using Monte Carlo simulated digital patient phantoms

**H. Pieters^1,2^, J. Van Staden^2^, H. Du Raan^2^**

^1^ *Klerksdorp Hospital, Klerksdorp, South Africa*

^2^ *University of the Free State, Bloemfontein, South Africa*

#### Background

Left ventricular ejection fraction (LVEF) is an important parameter for evaluating cardiac function. Commercial software programs are used to calculate LVEF values from planar gated blood pool (GBP-P) studies acquired with gamma cameras in nuclear medicine. However, before implementing these programs in clinical practice, it is essential to validate the accuracy and precision of the LVEF values obtained with these software programs using phantom studies. The aim of this study was to assess the accuracy of LVEF values obtained with a commercial software program using gamma camera-simulated GBP-P studies of digital patient models.

#### Methods

The SIMIND Monte Carlo program, along with the 4D-XCAT (XCAT) phantom, was utilised to simulate 69 male and female GBP-P studies with different combinations of LVEF values (19.9% – 77.4%) and end-diastolic volumes (86 mL–155 mL). These studies were then converted to DICOM format and transferred to a processing station with the available commercial software program, ALPHA NUCLEAR (AN). The ‘known’ LVEF of the XCAT phantom, determined from the exact volume of the left ventricle in the end-diastolic and end-systolic positions, was considered the gold standard. Processing was performed by three independent, experienced operators, and LVEF values were reported. Intra- and inter-variability were evaluated using the interclass correlation coefficient (ICC). A regression analysis was performed to compare the real LVEF and processed LVEF values.

#### Results

The intra-observer variability for all three observers was excellent (ICC1 = 0.99; ICC2 = 0.99; ICC3 = 0.98). The ICC evaluating the interobserver variability was 0.98. When comparing the average LVEF obtained by the three observers using the AN software, with the known XCAT LVEF, a good correlation was obtained (*r*^2^ = 0.94). A slight underestimation of the XCAT values at lower LVEF values was seen (LVEFAN = 1.09; LVEFXCAT – 7.59).

#### Conclusion

Independent processing of GBP-P studies using AN software indicated excellent intra- and inter-observer variability. A good agreement was found between the calculated LVEF values using the AN software and the known LVEF of the XCAT simulated images. The digital patient models created with the 4D-XCAT phantom provided a database of studies with known LVEF values whereby AN commercial software was validated. This study will be expanded by evaluating different commercial systems using the same simulated GBP-P studies.

### Estimation of radiation dose to commuters using patient transport and the nursing staff from thyrotoxicosis patients treated with radioactive iodine-131 at Universitas Academic Hospital

**M. Mongane^1,2^, T. Trinder-Smith^1^, K. Gabayo^1^**

^1^ *Universitas Academic Hospital, Bloemfontein, South Africa*

^2^ *University of the Free State, Bloemfontein, South Africa*

#### Introduction

Thyrotoxicosis patients are treated with radioactive I-131 to control the disease. The challenge comes after the I-131 treatment. The patient needs to be accommodated in a single room and separate rooms as per SAHPRA regulations, public dose below 1 mSv/year. The patients treated at Universitas Academic Hospital (UAH) come from the Free State, Eastern Cape, Northern Cape and Lesotho. As a result of prevailing economic disparity after discharge, some patients may thus be obliged to travel long distances (4–7 h) in crowded public transportation (e.g. FS Health Patient Transport Services). The ongoing concern of the dose to nursing staff caring for these patients was also addressed.

#### Aim

The study aimed to determine the 24 h radiation exposure from thyrotoxicosis patients’ post-treatment with I-131.

#### Methods and materials

Two electronic alarm dosimeters were used to measure radiation exposure in the room of admitted I-131 patient, and a total of 85 patients were monitored (October 2021 – December 2022). The dosimeter was at the wall (head side of the patient bed), 1.5 m above the floor, and 0.2 m from the patient’s head. The second set of measurements was performed at the wall (feet side of the patient bed), 1.5 m above the floor, and 2 m from the patient’s abdomen. The average time to monitor the room’s radiation levels was approximately 18 h – the mean prescribed I-131 activity was 15.7 ± 2.9 (ranging from 12 mCi to 20 mCi).

#### Results and discussion

The results show that radiation dose in the room varied from 106 µSv to 6220.0 µSv (Median: 385.0 µSv, Mean: 545.0 µSv ± 793.0 µSv) for approximately 18.6 ± 6.9 h post I-131 administration. The results indicate that the I-131 patients might give significantly higher radiation doses to other passengers using patient transport because of the reduced distance between them. The results for the second part show that radiation dose in the room varied from 32.0 µSv to 149.0 µSv (Median: 57.0 µSv, Mean; 66.8 µSv ± 35.5 µSv) for approximately 18.6 ± 2 h after I-131 administration.

#### Conclusions

The SAHPRA regulation of isolating patients after the I-131 therapy capsule ensures that the family and public are safe from radiation exposure. The dose received by the nursing staff is low. However, the dose to passengers in patient transport can be higher if patients are treated as outpatients without considering socioeconomic factors.

### Managers’ perspectives of service delivery in public sector nuclear medicine departments in South Africa

**A. Ndlela^1^, J. Thambura^2^, H. Essop^3^**

^1^ *Dr George Mukhari Hospital, Orchards, South Africa*

^2^ *University of Pretoria, Pretoria, South Africa*

^3^ *University of Pretoria, Pretoria, South Africa*

#### Introduction

Nuclear medicine (NM) is a rapidly growing discipline. Across SA, there are 14 and 46 NM departments in the public sector and private sector, respectively. Most SA public sector hospitals are strained because of a lack of adequate resources resulting in delayed bookings and patient waiting lists because of fewer examinations being performed daily. Service contracts between private and public hospitals as a solution for patient access to NM come at a high cost and prove ineffective in curbing the number of unassisted patients.

This research aimed to assess the service delivery of NM departments in the public healthcare system from the managers’ perspectives.

#### Methodology

This is qualitative research that employed the exploratory research design. The data were collected using a structured interview mostly conducted telephonically. In this research, 11 out of 14 managers in the 14 NM departments from five provinces in SA participated. Interviews were mainly open-ended and centred on addressing shortcomings perceived in ensuring adequate service delivery to public sector NM departments.

#### Results

Three themes pertaining to service delivery in NM emerged, namely barriers affecting service delivery, enablers that influence service delivery, and recommendations to improve service delivery. The majorly devastating findings were on barriers affecting service delivery. These comprised five categories, namely poor service delivery resulting from the unavailability of different resources, poor working conditions, the effects of the COVID-19 pandemic, inadequate management and administration and limited access to NM departments, especially in rural communities. Accolades accomplished by different NM departments were illustrated as enablers. Hard-working departments provided quality NM services for the population and had personnel willing to go the extra mile despite the observed barriers. The recommended solutions were to improve what is available, decentralise services to rural communities, allow personnel who work in the field to implement some of the advances in NM and use of technology and training to improve the services of the NM departments.

#### Conclusion

It was evident from the study that barriers have a devastating impact on service delivery. The unavailability or the lack of key resources was regarded as a major cause of discomfort for healthcare workers and patients. These limitations impact negatively on NM in the clinical setting as a theragnostic department and lead to suboptimal service delivery. There is a lot to be performed at all levels of the healthcare system in order to optimise service delivery on NM in the public healthcare sector.

### Validation of an in-house developed therapeutic dosimetric software tool for the treatment of 177lutetium-dotatate peptide receptor radionuclide therapy

**B. Van Wyk^1^, F. Hasford^2^, N. Nyakale^1^, M. Vangu^3^**

^1^ *Sefako Makgatho University, Pretoria, South Africa*

^2^ *Ghana Atomic Energy Agency, Accra, Ghana*

^3^ *University of the Witwatersrand, Johannesburg, South Africa*

Different computational software tools used in nuclear medicine have been written to improve radiation dose assessment, especially in therapeutic nuclear medicine. While many studies have investigated therapeutic nuclear medicine dosimetry, it has been noticed that very few articles compare the therapeutic software tools to each other. The aim of our study was therefore to validate our in-house developed software tool, Masterdose, using the commercial software OLINDA/EXM 1.0.

Methodology was based on clinical patient data treated for neuroendocrine tumours with 177Lutetium (Lu)-DOTATATE at a South African hospital. All patients underwent the same SPECT acquisition protocol and were corrected for scatter, partial volume, collimator-detector response, gamma camera calibration and attenuation. Correction factors (CFs) were applied to images to convert counts to activity. The first cycle of peptide receptor radionuclide therapy (PRRT) for 11 single photon emission computed tomography (SPECT) patients was compared on the Masterdose and OLINDA/EXM 1.0 software tools for 1, 24, 72 and 168 h. Cumulated radionuclide activity and the absorbed dose were compared for the two software tools. The absorbed dose difference was then compared using statistical Bland-Altman analysis.

Masterdose and OLINDA/EXM 1.0 had different peptide receptor radionuclide therapy methodologies. This led to different results obtained for the software tools. The OLINDA/EXM 1.0 software did not propose steps for image quantification. Therefore, Masterdose quantification results were compared with the Dosimetry Toolkit^®^ (DTK) on the Xeleris^®^ software by GE Healthcare. The largest cumulated activities were found in tumours compared with the kidneys for both software. On an average, the relative percentage difference (RPD) between the cumulated activities of Masterdose and DTK was 10.5% and 10.9% for the kidneys and tumours, respectively. Absorbed dose differences were similar in magnitude. For the kidney Bland-Altman analysis, a particular trend was seen; the calculated absorbed doses were almost consistently higher than the bias or systematic error of 0.2 Gy. However, the tumour Bland-Altman analysis shows a non-systematic difference, as calculated absorbed doses were distributed evenly around the bias of 1.9 Gy.

As a result of the different therapeutic approaches of the two software programs compared, results obtained during the comparison affected the results of this study. The RPD between the software was attributed to different methodologies during the CFs and activity quantification of Masterdose and OLINDA/EXM 1.0.

### Coronary [68GA] Ga NODAGAZOL PET versus coronary artery calcium scoring in the evaluation of atherosclerotic activity

**H. Ndlovu^1,2^, I. Lawal^2,3^, K. Mokoala^1,2^, G. Popoola^4^, T. Annor^5^, M. Sathekge^1,2^**

^1^ *Nuclear Medicine Research Infrastructure (NuMeRI), Steve Biko Academic Hospital, Pretoria, South Africa*

^2^ *Department of Nuclear Medicine, University of Pretoria and Steve Biko Academic Hospital, Pretoria, South Africa*

^3^ *Department of Radiology and Imaging Sciences, Emory University, Atlanta, Georgia, United States of America*

^4^ *Saxon Court Lincolnshire Partnership NHS foundation trust (LPFT), Lincoln, United Kingdom*

^5^ *Department of Radiology, University of Pretoria and Steve Biko Academic Hospital, Pretoria, South Africa*

#### Introduction

Coronary artery calcium (CAC) scoring is a reliable method for cardiovascular risk stratification and predicting outcome in patients with coronary atherosclerotic disease (CAD). [68Ga]Ga-NODAGAZOL positron emission tomography/computed tomography (PET/CT) is a novel molecular technique for imaging microcalcification: a hallmark of atherosclerotic plaque instability. In our previous work, we demonstrated a positive correlation between [68Ga] Ga-NODAGAZOL uptake and the cardiovascular risk profile of patients. The aim of this study was to establish the relationship between [68Ga] Ga-NODAGAZOL uptake and CAC score in atherosclerotic plaque and to assess the cardiovascular outcome.

#### Methods

A total of 18 patients with CAD were prospectively recruited to undergo cardiac computed tomography (CT) and [68Ga] Ga-NODAGAZOL PET/CT within 2 weeks of each other. The CAC score for each of the three coronary arteries was computed. The patients were followed-up for major cardiovascular events including death, non-fatal myocardial infarction, or new-onset angina. [68Ga] Ga-NODAGAZOL uptake in the atherosclerotic plaques in the respective arteries was quantified using the maximum standardised uptake (SUVmax). For each artery, the atherosclerotic plaque with the highest SUVmax was used as the representative lesion for the respective artery. The SUVmax was correlated to the CAC score using the non-parametric Spearman-rank test, whereas the Mann–Whitney *U* test was used to determine the correlation of CAC and [68Ga] Ga-NODAGAZOL uptake with cardiovascular outcome.

#### Results

Ninety-four per cent of patients had BMI > 25 kg/m^2^ (94%) whereas 67%, 56%, 72% and 61% had a history of dyslipidaemia, systemic hypertension, diabetes mellitus and smoking, respectively. The patients tolerated the [68Ga] Ga-NODAGAZOL with no untoward side effects. The mean injected activity [68Ga]Ga-NODAGAZOL was 144.67 MBq (range = 92.5 – 244.2). The median (range) CAC score was 118.3(1.1–1326.1). The SUVmax of [68Ga] Ga-NODAGAZOL uptake in LAD, RCA, and LCx arterial territory were as follows: median (range): 2.05 (1.56–3.34), 2.05 (1.33–3.41) and 1.23 (1.06–2.75), respectively. Coronary artery calcium score demonstrated a strong positive correlation with the SUVmax of [68Ga]Ga-NODAGAZOL uptake *r* = 0.545 (*p* < 0.001). Coronary artery calcium score of the different arterial territories also showed significant correlation to cardiovascular outcome (LAD, *p* = 0.011; RCA, *p* = 0.021; and LCx, *p* = 0.013). Similarly, [68Ga] Ga-NODAGAZOL uptake measured using SUVmax showed significant correlation with cardiovascular outcome in the LAD (*p* = 0.026) and RCA (*p* = 0.021) but not in the LCx (*p* = 0.052) arterial territories.

#### Conclusion

There is a strong positive connection between CAC score and the degree of coronary [68Ga] Ga-NODAGAZOL uptake in atherosclerotic plaques in a CAD cohort. Furthermore, both CAC and [68Ga] Ga-NODAGAZOL uptake exhibit a strong positive correlation with long-term cardiovascular outcomes.

### Application of microPET-CT to in-vitro computations

**C. Swanepoel^1^, M. Visagie^2^, C. Driver^1,3^, J. Van Laar^2^, L. Jordaan^2^, T. Ebenhan^1,3^**

^1^ *Numeri Npc, Pretoria, South Africa*

^2^ *Department of Physiology, Faculty of Health Sciences, University of Pretoria, Pretoria, South Africa*

^3^ *Department of Radiochemistry, South African Nuclear Energy Corporation, Hartbeespoort, South Africa*

Clinical positron emission tomography-computed tomography (PET-CT) is routinely used for cancer staging and therapeutic response. Preclinical evaluation of novel radiopharmaceutical compounds requires in vitro cancer cell studies with PET-CT investigations for imaging of cancer cells that have been attempted.^1^ However, higher system resolution is needed for in vitro tests. A microPET-CT (µPET-CT) offers adequate resolution; however, there is a lack of literature on the use of this modality for in vitro application. Acquisition parameters and methodology need to be established to provide a validated method for using µPET-CT when imaging cell plates.

A µPET/CT scanner with associated accessories (carbon fibre plate, 22Na-point source and post-processing software) along with an automated gamma counter was utilised for the in vitro analysis. Cancer cells were cultivated in a six-well plates to a suitable starting density. 18F-fluorodeoxyglucose (18F-FDG) (1.8 MBq ± 0.3 MBq) was administered into each well and was left for a 60 min incubation period. Each well was subjected to three wash cycles using phosphate buffer solution (PBS) to remove the excess media and unbound 18F-FDG. Each rinsed solution was counted on the gamma counter and a standard dilution of 18F-activity was created as a reference for activity quantification. Imaging was commenced immediately following the last wash cycle using µPET/CT. Following the µPET/CT acquisition, the cells were scraped from each well, collected in PBS and the activity was measured using the gamma counter. A field of view containing the 6-well plate was reconstructed and the volume of interests associated with each well was quantified. 18F-FDG-image guided uptake/well was verified by sample gamma counting.

The 18F-FDG-µPET/CT imaging of cells was successful, i.e. sufficient dose yielded a good count rate and signal-to-background ratio. Tracer uptake in cancer cells was detected and further used to quantify normalised uptake values (kBq/mL), which correlated well with the 18F-FDG signal in the cell pellet by using gamma counting. The acquisition method was also optimised over a series of acquisitions to ensure future acquisitions show minimal variances.

The acquisition parameters and methodology for in vivo imaging using µPET/CT were established. Therefore, this study is precedent that the use of µPET/CT imaging is not limited to in vivo procedures but can also be successfully applied to an in vitro setting. This will further streamline the translation of pre-clinical research to the clinical field.

### Application of single SPECT/CT and Cerenkov luminescence for imaging of therapeutic isotopes

**C. Swanepoel^1^, C. Driver^1,2^, T. Ebenhan^1,2,3^**

^1^ *Numeri Npc, Pretoria, South Africa*

^2^ *Department of Radiochemistry, South African Nuclear Energy Corporation, Hartbeespoort, South Africa*

^3^ *Department of Nuclear Medicine, University of Pretoria, Pretoria, South Africa*

Therapeutic radionuclides are not easily visualised and are paired with positron (β+) or gamma-emitting radionuclides to evaluate disease progression using conventional nuclear imaging techniques (positron emission tomography [PET]; single photon computed tomography [SPECT]). Popular theranostic pairs include 68Ga/177Lu, 68Ga/255Ac, 64Cu/67Cu or 188Re/99mTc. Single photon computed tomography imaging of therapeutic isotopes is not routinely used because of the generally low yield in gamma emissions of these isotopes. However, technological advances in SPECT imaging hardware and software have made imaging of these tracers more feasible.

Cerenkov-derived luminescence imaging (CLI) detects light emitted because of charged particles from radioactive decay moving faster than light in a medium; therefore, CLI is suitable to directly visualise beta-emitting therapeutic radionuclides. This aspect should be utilised more to support clinically relevant questions. This study evaluated SPECT and CLI-capabilities of three, relatively unused, therapeutic nuclides (186Re, 188Re, 109Pd) in comparison to already evaluated therapeutic nuclides (177Lu,131I) and diagnostic (68Ga, 64Cu, 18F, 89Zr) nuclides for application of theranostic radiopharmaceuticals.

Single photon computed tomography/computed tomography – each isotope was calibrated according to manufacturer specifications using a multi-pinhole collimator. Images for all radionuclides were acquired using Derenzo and PET National Electrical Manufacturers Association (NEMA) phantoms. Following image reconstruction, the images were evaluated according to: sensitivity, resolution, uniformity and contrast signal-to-noise ratio (SNR).

Cerenkov-derived luminescence imaging – A 1:2 dilution series of each radionuclide (1 mL in saline) was pipetted into a 24-well plate (activity 0–9.16 MBq/mL). Each radionuclide underwent CLI at half-life appropriate time points. Radionuclides were compared with respect to activity-concentration (kBq/µL) and average radiance (p/s/cm^2^/sr).

Single photon computed tomography/computed tomography – Isotopes with gamma rays in the vicinity of 140 keV showed the best sensitivity and resolution (188Re and 186Re); visual uniformity of Rhenium-188 was superior to Rhenium-186. Compared to 177Lu and 131I evaluation of the SNR of 186Re- and 188Re-isotopes and 109Pd correlated as expected.

Cerenkov-derived luminescence imaging – Linear regression and range analysis for all nuclides indicated a strong, positive correlation between the light emission intensity and activity concentration (*R* = 0.99). High energy emissions of 188Re (Eβmax = 2.10 MeV; Eβ(ave) = 0.8 MeV; 70% intensity) corresponded to radiance observed for 68Ga (Eβmax = 1.89 Mev; Eβave = 0.8 MeV; 87% intensity) while radiances for 109Pd and 186Re were 2.5- and 5-fold lower, respectively (all other nuclides ≤15-fold lower).

Rhenium-186/-188 exhibits superior SPECT imaging capabilities over 131I- and 177Lu-radionuclides while 109Pd still provides suitable SPECT images. All three therapeutic isotopes have excellent CLI properties comparable to other therapeutic-/PET-isotopes. The SPECT- and-CLI imaging capabilities of these therapeutic radionuclides allow for further research into more comprehensive targeting mechanism.

### Preclinical PET/CT as imaging modality to showcase the effect of chemical properties on overall in vivo biodistribution

**C. Swanepoel^1^, C. Driver^1,2^**

^1^ *Numeri Npc, Pretoria, South Africa*

^2^ *Department of Radiochemistry, South African Nuclear Energy Corporation (Necsa), Hartbeespoort, South Africa*

Radiopharmaceuticals for diagnostic imaging through positron emission tomography/computed tomography (PET/CT imaging) are designed to target specific diseases. The design of these radiopharmaceuticals during the drug development process focusses on chemical features such as stereochemistry, steric hindrance, molecular modelling and structure-activity relationship. However, when it comes to application of these compounds in vivo, there are additional factors that affect the compound’s biological distribution, excretion and targeting efficacies, which need to be considered.^1^ These chemical properties include: molecular weight, lipophilicity and overall charge (if applicable) of a compound.

The aim of this retrospective study was to compare the microPET/CT images of seven selected radiopharmaceuticals and correlate the visualised biodistribution with the drug’s chemical properties.

The radiopharmaceuticals were formulated in saline or 0.1 M PBS with pH 7–7.4 and administered intravenously into the mouse tail vein (volume < 200 µL and activity dose of 8–10 MBq per animal). Mice were imaged over 20–30 min in list mode using microPET/CT. Timepoints for imaging were 1 h, 2 h, 4 h, and 24 h dependent on the radionuclide used and its corresponding half-life. Images were reconstructed iteratively with attenuation and activity correction to allow for quantification.

The visualised biodistribution and excretion of the following radiopharmaceuticals: 18F-FDG, 68Ga- prostate-specific membrane antigen (PSMA)-11, 64Cu-TETA, 64Cu-GluCAB2-NH2, 89Zr-DFO-SC, 64Cu-GluCAB1-Mal-HAS and 89Zr-DFO-IIIB6 were compared, and the results were correlated to the compounds molecular weight, lipophilicity and overall charge.

The metabolism and excretion of the radiopharmaceuticals follow either a renal (kidney) or hepatobiliary (liver, intestines) pathway. Smaller compounds (under glomerular filtration size) that are generally negatively charged with a less lipophilic structure follow a renal excretion route while larger compounds (over glomerular filtration size) that are often protein/antibody bound, generally positively charged and more lipophilic, exhibit slow clearance through a hepatobiliary pathway. Exceptions were observed when desired biodistribution effect based on compound size was overshadowed by the charge and lipophilicity of the compound.

Different radiopharmaceuticals present with different biodistribution and excretion patterns that can be effectively visualised using microPET/CT. The chemical properties (molecular weight, net charge and lipophilicity) of a radiopharmaceutical affect the biodistribution and subsequent excretion pathway and therefore need to be considered when designing new radiopharmaceuticals.

### Scrotal wall pertechnetate washout as a predictor of testicular salvage for missed torsion by testicular scintigraphy

**N. Zondi-Mredlana^1,3^, X. Khuzwayo^1,3^, W. Pilloy^1,3^, S. Mutambirwa^2,3^, N. Nyakale^1,3^**

^1^ *Department of Nuclear Medicine, Dr George Mukhari Academic Hospital, Pretoria, South Africa*

^2^ *Department of Urology, Dr George Mukhari Academic Hospital, Pretoria, South Africa*

^3^ *Sefako Makgato Health Science University, Pretoria, South Africa*

#### Background and objective

Testicular torsion (TT) is a urological emergency; early diagnosis and intervention prevent long-term complications, including male infertility. In our case series, we aimed to distinguish if the washout of tracer from the scrotum could be predictive of salvageability of the testes in patients with testicular scintigraphy (TS), which is diagnostic of testicular torsion.

#### Methods

We evaluated the TS scans of patients who underwent surgical intervention for orchidectomy for suspected missed TT from January 2016 to December 2021 at Dr George Mukhari Academic Hospital. We assessed the washout of tracer in the scrotum between 5-min and 20-min images and determined the correlation with salvageability of the testes in patients scintigraphically diagnosed with missed TT. Semiquantitative (maximum and minimum counts) and visual analysis were conducted.

#### Results

A total of 12 patients underwent surgery. Of these, 50% of the testes were salvaged and in 50%, the testes could not be salvaged and complete orchidectomy was performed. On visual and semiquantitative analysis, we detected a progressive decrease in pertechnetate uptake in the scrotal wall uptake in the salvaged testes. The maximum percentage of decrease in counts in this group was 34%; the median time to presentation after the onset of symptoms was 2.5 days with an interquartile range of 2 days and a range of 1 day to 5 days. In the non-salvaged group, visual and semiquantitative increase in uptake in scrotal wall was observed. The maximum percentage increase in counts was 32%. The median time to presentation after the onset of symptoms in these patients was 6 days with an interquartile range of 5 days and a range of 2 days to 14 days.

#### Conclusion

This case series demonstrates an association between pertechnetate washout and salvageability of the testes. Testicular salvage is observed in patients who demonstrated a progressive decrease in uptake of pertechnetate in the scrotal wall over a 20 min imaging time and in patients who presented beyond the golden 6 h time limit. Testicular scintigraphy may be a great tool in identifying those patients with higher possibility of salvage and assist to prioritise their surgery.

### Systematic assessment of anaesthetic procedures for better alignment and compliance with radiotracer microPET-CT imaging in small animals

**J. Visser^1,2^, C. Swanepoel^1^, C. Driver^1,2^, T. Ebenhan^1,2,3^**

^1^ *Numeri Npc, Pretoria, South Africa*

^2^ *NECSA – Radiochemistry, Hartbeespoort, South Africa*

^3^ *Nuclear Medicine Department, University of Pretoria, Pretoria, South Africa*

Non-invasive, functional imaging techniques such as positron emission tomography-computed tomography (PET/CT) provide the means for studying physiological and pharmacological processes in real-time, both in animals or humans (1) Positron emission tomography-computed tomography imaging has made a major footprint in preclinical research of pharmacology, molecular biology and medical sciences, facilitating innovation in neuroscience, cancer, inflammation and cardiovascular research. Rodent animal models are commonly anaesthetised with ketamine or isoflurane in order to prevent movement artefacts during image acquisitions (2) however, such anaesthesia often alters the physiological condition of the rodents. Consequently, the micro positron emission tomography/computed tomography (microPET/CT) findings obtained in anaesthetised animals may not accurately represent the physiological properties (3) This investigation aims to systematically align different anaesthetics with common radiotracers for PET/CT imaging studies to provide information that can enhance the radiotracer representation according to their targeting mechanism in rodents to advance the quality and translatability of microPET/CT-derived data.

The following compatible anaesthetics were not only mainly assessed for suitability of using [18F]fluorodeoxyglucose ([18F]FDG) but also for [68Ga] Ga-DOTA-tate-, or [68Ga]Ga-PSMA-11-microPET-CT diagnostics: isoflurane, sevoflurane, pentobarbital, propofol or combination of fentanylcitrate-fluanisone/diazepam and ketamine/xylazine. These anaesthetic agents were compared based on animal physiology (respiration rate, body temperature, apparent glucose metabolism and expected radiotracer biodistribution).

The investigated anaesthetics caused altered respiration rate, body temperature, glucose metabolism and have effects on target organs, which affected radiotracer biodistribution. Isoflurane inhalation is the most desired anaesthetic procedure; however, both sevoflurane and isoflurane (also ketamine/xylazine administration) induced hypoglycaemia. Sevoflurane can be suggested over isoflurane for cardiac imaging. Propofol is suitable for [18F]FDG imaging but should be avoided for neuroimaging causing decreased cerebral glucose metabolism. Pentobarbital does not alter the blood glucose metabolism but has a lower safety margin for rodent anaesthesia. Fentanylcitrate-fluanisone/diazepam the preferred injectable anaesthetic option for [18F]FDG being the least interfering with glucose metabolism. Tumour imaging quality using [68Ga]Ga-DOTA-tate- or [68Ga]Ga-PSMA-11-microPET/CT was supported by isoflurane or sevoflurane; however, higher glucocorticoid levels and certain neuroendocrine abnormalities occurred (may originated by hypothermia).

This investigation proved that anaesthetics used for preclinical research using microPET/CT imaging need to be carefully aligned with the radiotracer’s half-life, and metabolism, to warrant an uninterrupted image acquisition of high quality. A better understanding of the interplay between radiotracers and available anaesthesia agents can allow for the best choice of anaesthesia that will improve the performance and outcome of imaging studies. Thus, radiotracers are supported to show their full research potential to become non-invasive in vivo biomarkers or sensitive drug efficacy monitoring tools.

### Individualised calibration of estimated glomerular filtration rate for serial renal function monitoring

**B. Mekonnen^1^, J. Holness^1^, B. Berndorfler^1^, J. Warwick^1^**

^1^ *Stellenbosch University, Cape Town, South Africa*

#### Background

Glomerular filtration rate (GFR) is widely accepted as the best index of renal function. Estimated GFR (eGFR) calculated by serum creatinine remains the most widely used method in clinical practice. However, eGFR lacks precision and accuracy in early stages of kidney disease (stages 1 and 2) and is not a reliable measure of change in GFR over time. Therefore, in certain groups of patients where decisions based on eGFR may have significant clinical consequence, measurement of GFR is recommended. However, the currently used methods to measure GFR are comparatively expensive and time-consuming and are not widely available. As a compromise between measured and estimated GFR, particularly for patients who require serial GFR measurements, it is possible to develop individualised eGFR equations by calibrating the first GFR estimate using an initial GFR measurement taken at the same time. By applying an individualised calibration factor to subsequent estimates, it may be possible to provide an improved individualised estimate of renal function. This would be beneficial in settings where there is limited access to GFR measurement.

#### Methods

Patients who had undergone two radionuclide-based GFR measurements within 3 months of serum creatinine measurement at the nuclear medicine division, Tygerberg Hospital, between January 2009 and December 2019 will be retrospectively identified. The 2021 CKD-EPI Creatinine equation will be used to calculate the estimated GFR. A patient-specific calibration factor (Kpt) will be calculated from the initial measured (mGFR1) and initial estimated GFR (eGFR1), where mGFR1 = Kpt*eGFR1, i.e. Kpt = mGFR1/eGFR1. For subsequent studies, a patient-specific eGFR (eGFR-Pti) will be calculated using the patient-specific calibration factor and the estimated GFR at that particular time (eGFRi = Kpt*eGFRi). Bland-Altman analyses will be used to compare eGFR and patient-specific eGFR to measured GFR.

#### Results

Selected patients will be described in terms of their demographic profile, clinical indication, and relative timing of creatinine and GFR measurements. The results of Bland-Altman analyse the performance of patient-specific eGFR (eGFR-Pti) to conventional eGFR in terms of bias and limits of agreement.

#### Conclusion

Individualised calibrated GFR is expected to perform better than estimated GFR in serial renal function monitoring. This would provide a practical alternative to serial GFR measurement in environments where there is limited access to GFR measurement.

### Should fluorodeoxyglucose positron emission tomography integrated with computed tomography replace methylene diphosphonate bone scintigraphy in the management of breast cancer patients with suspected skeletal metastases?

**M. Patel^1^, M. Vorster^1^, B. Hadebe^1^, V. Pillay^1^, L. Harry^1^, L. Gabela^1^, S. Masikane^1^, T. Nxasana^1^**

^1^ *University of KwaZulu-Natal, Durban, South Africa*

Breast cancer is the leading cause of cancer-related death in women and the fifth leading cause of mortality worldwide. Bone metastases is the most frequent site of dissemination. Prevention of morbidity from metastases to bone requires accurate, early diagnosis for preliminary staging, planning of treatment, treatment monitoring, restaging and prediction of survival in patients with breast cancer. Many centres in South Africa still mostly make use of 99m Tc-MDP bone scintigraphy in the staging work-up of these patients, likely because of the relatively low cost, availability, and physician familiarity with this imaging modality. 18F-fluorodeoxyglucose positron emission tomography/computed tomography (18F-FDG PET/CT) scans are more sensitive in detecting lytic lesions and purely marrow involvement. Additional soft tissue involvement can also be detected using this imaging modality. The purpose of this cross-sectional study is to compare the detection of suspected metastatic skeletal lesions in both 99m Tc-MDP bone scintigraphy and 18F-FDG PET/CT scans as well as its impact on management.

#### Methods

Forty-six breast cancer patients with a mean age of 52 years underwent a 18F-FDG PET/CT scan and a 99m Tc-MDP bone scintigraphy performed within 31 days between January 2017 and March 2023. Comparison was performed based on a lesion-by-lesion analysis. The results of the image interpretation were compared retrospectively. Any change in stage and management was recorded.

#### Results

A total of 142 lesions were detected on 99m Tc-MDP bone scintigraphy and 237 lesions were detected on 18F-FDG PET/CT scans, which led to a change in management of 12 patients with 99m Tc- MDP bone scintigraphy and 17 patients with 18F-FDG PET/CT scans. Of the 46 patients, 76% (*n* = 35) of patients had invasive ductal carcinoma (IDC), 13% (*n* = 6) had invasive breast cancer no specific type 4(IBC-NST), 4.3% (*n* = 2) had invasive lobular cancer (ILC) and 6.5% (*n* = 3) of patients had missing histological results. In 1/46 (2%), an osteoblastic lesion was detected on the 99m Tc-MDP bone scintigraphy and negative on 18F-FDG PET/CT scan, which resulted in change in management. This patient had triple-positive IDC. With regard to tumour stage (T), 63% (*n* = 29) had T4 lesions, 17% (*n* = 8) had T3 lesions, 15% (*n* = 7) had T2 lesions and 4% (*n* = 2) had T1 lesions. Detailed statistical analysis to follow.

#### Conclusion

18F-Fluorodeoxyglucose positron emission tomography/computed tomography scans proved to be the superior modality in detecting skeletal metastases and other soft tissue distant metastases resulting in a significant number of patients having a change in management in comparison to 99m Tc-MDP bone scintigraphy.

### Perceptions of nuclear medicine technologists on PET/CT in Gauteng Province, South Africa

**L. Manzana^1^, L. Chipeya^2^, P. Bresser^3^**

^1^ *University of Johannesburg, South Africa*

^2^ *University of Johannesburg, South Africa*

^3^ *University of Auckland, New Zealand*

In South Africa, the first positron emission tomography/computed tomography (PET/CT) imaging modality was installed in Gauteng in 2005 with subsequent installations. The introduction of PET/CT requires appropriate staff training, redesign of patient workflow, new skills, problem-solving abilities and radiation protection. With the introduction of PET/CT in the UK, nuclear medicine technologists (NMTs) encountered challenges in defining their roles, unfamiliarity with the new technology, and new working procedures, which led to confusion and concerns about the new technology.

Since the introduction of the first PET/CT in South Africa, the perceptions of NMTs of this dual imaging technique have not been determined. Therefore, this research study aimed to explore and describe the perceptions of NMTs regarding PET/CT as an imaging modality in nuclear medicine. The objectives formulated to achieve the aim were to establish how NMTs in Gauteng perceive PET/CT as an imaging modality and to make recommendations on managing the needs and desires of NMTs based on study’s findings.

A qualitative, descriptive phenomenological research design was applied with semi-structured interviews until data saturation was reached. Purposive sampling was employed to select 18 practising NMTs in Gauteng to participate in this study. The participating NMTs consisted of three cohorts: those with PET/CT experience, those with no PET/CT experience, and those with previous PET/CT experience but not exposed to PET/CT at the time of data collection. Thematic analysis was used to facilitate the management of codes, categories and themes during the analysis of the data.

The three themes that emerged from the categories are NMTs’ perspectives of PET/CT in nuclear medicine, patient management in PET/CT, and PET/CT challenges. The findings suggest that NMTs in Gauteng perceived PET/CT as the future of nuclear medicine. They acknowledged the positive role played by PET/CT in patient management, especially oncology patients. Findings also reveal a gap in PET/CT training in Gauteng, which the NMTs perceived as hindering their career growth in nuclear medicine. Nuclear medicine technologists raised concerns regarding high radiation exposure associated with PET/CT imaging, a lack of psychological support, single qualification in nuclear medicine, and a lack of PET/CT exposure. Based on the study’s findings, recommendations are made to manage the needs and desires of NMTs in Gauteng. Recommendation for PET/CT training collaboration between the government and private nuclear medicine departments to facilitate CPD-accredited rotation clubs between nuclear medicine departments.

### Assessment of the longitudinal radiolabelling performance of kit-based gallium 68 prostate-specific membrane antigen 11 preparations to support theranostics of prostate cancer

**L. Mohlala^1^, M. Lundie^1^, J. Mwazembeni^1^, T. Ebenhan^2^**

^1^ *Sefako Makgatho Health Sciences University, Pretoria, South Africa*

^2^ *Sefako Makgatho Health Sciences University, Pretoria, South Africa*

^3^ *Sefako Makgatho Health Sciences University, Pretoria, South Africa*

^4^ *South African Nuclear Energy Corporation, Pelindaba, South Africa*

#### Introduction

Prostate-specific membrane antigen (PSMA) is overexpressed in prostate cancer (PC) and has been discovered as a target for theranostics in nuclear medicine. Therapy response can be monitored using positron emission tomography/computed tomography (PET/CT) imaging using [68Ga]Ga-PSMA-11, a peptide ligand that can be conveniently prepared from freeze-dried kit starting material currently developed at the South African Nuclear Energy Corporation. This study aimed investigating kit-based [68Ga]Ga-PSMA-11 preparations from a large sample size (*n* > 350). Variations observed within radiolabelling efficiencies, radiochemical purities and yields provided the input assessing the longitudinal radiolabelling robustness and helped determining those parameters and factors leading to lower radiopharmaceutical yields or preparation failures.

#### Methods

An assessment of 388 [68Ga]Ga-PSMA-11 preparations over a 1-year period using kit starting material was conducted as a retrospective and cross-sectional study. In total, 330 data sets (85.0%) were enrolled in the analysis of the robustness of this labelling technique and also to identify the factors that may cause variations and failure of [68Ga]Ga-PSMA-11 preparations. Systematic evaluation of the responsible issues was addressed to enhance future radiopharmaceutical preparations.

#### Results

A total of 196/330 (59.4%) successful [68Ga]Ga-PSMA-11 preparations were achieved (i.e. a radiochemical purity ≥ 95%); of those, a 52.1% instant preparation (no cartridge purification required) and 7.3% failed rate occurred. For 134 (40.6%) preparations with a radiochemical purities 51% – 95% and for those yielding 45% – 50%, the purification process restored appropriate [68Ga]Ga-PSMA-11 product for 73.1% and 11.2% of the preparations, respectively. The level of training to operating personnel was correlated with the preparation success rate. The ideal radiolabelling reaction pH was 4.5, otherwise causing decrease in labelling efficiency. Adsorbed radioactivity to cartridge material was the main culprit for low radiochemical yields.

#### Conclusion

The performance of [68Ga]Ga-PSMA-11 preparation from kit starting material is easily applicable as a method with a low-cost technique that yields a high-quality product, ideal to be used in radiopharmacy environments with limited resources. It was proven that for most preparations with compromised purity, the product quality was restored by a Sep-Pak cartridge-based purification. Future [68Ga]Ga-PSMA-11 preparation success may be further increased by generator quality management, attention to expertise, and correct pH and Sep-Pak cartridge adjustments.

### 177Lu-PSMA radioligand therapy in the post-androgen deprivation therapy setting (prior to chemotherapy) in metastatic prostate cancer patients

**L. Maserumule^1^, K. Mokoala^1,2^, M. Sathekge^1,2^**

^1^ *Department of Nuclear Medicine, University of Pretoria, Pretoria, South Africa*

^2^ *NuMeRI and Steve Biko Academic Hospital, Pretoria, South Africa*

Prostate cancer remains a leading cause of mortality despite multiple therapy options. Peptide radioligand therapy (PRLT) with 177Lutettium-prostate specific membrane antigen (177Lu-PSMA) prolongs survival and improves quality of life in heavily pre-treated patients with metastatic castrate-resistant prostate cancer (mCRPC). Earlier introduction of PRLT in chemotherapy-naïve mCRPC patients is still not extensively explored.

#### Aim

To report the initial experience of PSA response and progression-free survival (PFS) in chemotherapy-naïve mCRPC patients treated with 177Lu-PSMA.

#### Methods

We retrospectively reviewed 18 chemotherapy-naïve patients with mCRPC who presented for PRLT. The patients received 177Lu-PSMA at 6–8 weeks intervals with an average activity 8 GBq per cycle. The PSA was measured prior of each cycle and monitored three months after completion of PRLT. The PSA response is classified as ≥ 50% decline, any decline and disease progression (≥ 25% increase above baseline).

#### Results

All patients had metastases on 68Ga-PSMA positron emission tomography/computed tomography (PET/CT). Seven and four patients had nodal and skeletal metastases, respectively; six had both and one had additional lung metastases. All patients had good functional status with Eastern Cooperative Oncology Group score of 0 to 1. All patients had received hormonal therapy, four had prostatectomy and seven had pelvic radiation. A total of 90 cycles (median 4, range 2–6) were administered. Following the first cycle, decline of ≥ 50% in PSA was observed in 22% and any decline in 61% of patients. Seven patients’ (39%) PSA increased after one cycle. After the last cycle, all 13 (72%) patients who had a PSA response demonstrated a decline of ≥50%. Ninety per cent of patients who had any PSA decline after the initial cycle had durable PSA response of ≥50%. Fifty-seven per cent of patients who had an initial increase in PSA progressed. All patients had a follow-up of at least 4 months. The median PFS was 9 months (range 4–36 months).

#### Conclusions

A positive response was observed in this chemotherapy-naïve cohort with durable PSA decline in over 70% of the patients and median PFS of 9 months after 177Lu-PSMA therapy.

### Changing the milk scan protocol, paradoxical or?

**K. Hlongwa^1^, N. Jacobs^1^, A. Al Nabulsi^1^, O. Kolade^1^, M. Jassen^1^, F. Smith^1^, A. Brink^1^, S. More^1^**

^1^ *University of Cape Town and Red Cross Children’s Hospital, Cape Town, South Africa*

#### Background/aim

Gastro-oesophageal reflux (GER) and pulmonary aspiration occur in children of all ages. Milk scans have been used for the detection of GER for decades. There are various documented protocols for milk scans in search of GER with no universally accepted protocol. We recently amended our protocol from imaging patients for 30 min and assessing gastric emptying at 2 h to imaging patients for 60 min and assessing gastric emptying at 3 h due to the potential of missing episodes of reflux and overclassifying delayed emptying when normal. This study aims to assess the value of the change in protocol in being able to detect significant number of refluxes.

#### Method

We retrospectively reviewed the scans of all patients who presented for milk scans at the Red Cross War Memorial Children’s Hospital from 01 November 2021 to 30 November 2022. The number of reflux episodes detected in patients imaged with the 30-min protocol in comparison to the number detected with the 60-min protocol was reviewed. The episodes of reflux were classified with an in-house severity scale and gastric emptying was evaluated at different time intervals. Delayed gastric emptying at 2 h was quantified as residual activity in the stomach > 37% and at 3 h as > 80% emptying into the bowel.

#### Results

A total of 200 studies were reviewed over the 13-month period (103 males and 97 females).

In the 30-min studies, 400 reflux episodes were observed in 70 patients, 7 were classified as normal, 9 mild reflux, 19 moderate reflux, 35 severe reflux, with 21 patients demonstrating delayed gastric emptying at the 2-h images. 11/21 patients with delayed gastric emptying had no reflux and 10/21 patients had reflux of varying classifications.

In the 60-min studies, 537 reflux episodes were observed in 65 patients, 16 were classified as normal, 8 mild reflux, 15 moderate reflux, 26 severe reflux with 12 patients demonstrating delayed gastric emptying at the 3-h images. 8/12 patients with delayed emptying had no reflux and 4/12 had reflux of varying classifications.

#### Conclusion

More episodes of reflux were detected in the 60-min reflux search without increasing incidence in patient numbers. Our findings did not demonstrate substantial differences owing to a change in the protocol. A standardised study comparing similar patient populations with the question of reflux may be beneficial in order to establish a common practice.

### Emerging radioisotopes; the production thereof and decay product considerations

**J. Zeevaart^1,2^, C. Driver^1,2^, Z. Szucs^1^**

^1^ *Necsa, Pretoria, South Africa*

^2^ *Nuclear Medicine Research Infrastructure NPC, Pretoria, South Africa*

There are a number of exciting new radioisotopes that are proposed, evaluated and produced in research quantities all claiming to be the best new radioisotopes for the future. Different aspects are highlighted: decay mode (alpha, beta, Auger), half-life, radiochemistry, production routes to name a few. In this article, radioisotopes will be evaluated in the light of the above but also in the particular South African setting with the view to the future multipurpose reactor (MPR) Necsa is planning to build to replace SAFARI-1 in the early 2030s.

Several of the new radioisotopes proposed do not consist of single decay and some have several daughters contributing to the radiation/therapeutic dose – also referred to as in vivo generators. The underlying physics and radiochemistry aspects of these will be presented and what it means for the chelators employed in the targeting vectors.

### De novo nuclear medicine services in the public health sector of North West Province: Turning vision into reality

**A. Ayeni^1,2^, H. Pieters^1^, T. Ramaloko^1^, M. Vangu^2^, B. Luke^1,2^**

^1^ *Klerksdorp/Tshepong Hospital Complex, Klerksdorp, South Africa*

^2^ *University of the Witwatersrand, Johannesburg, South Africa*

Klerksdorp/Tshepong (K/T) Hospital complex is a provincial tertiary hospital that serves the entire North West Province in South Africa. Commencement and expansion of hospital-based tertiary services in this health facility have been ongoing for the past two decades with many successes. Non-availability of nuclear medicine services in the public health sector of this province has resulted in patients being referred to academic hospitals in Gauteng with its attendant logistic and cost-related burden.

The journey of the first nuclear medicine clinical unit in the North West province public sector from conceptualisation to realisation culminated in its completion within the projected financial year. The implementation process had inevitable minor adaptations expected for a project of such complexity. However, this is an accomplishment that was made possible through strategic planning, creative problem-solving, and resourceful partnerships with relevant stakeholders. Funding was secured from NHI Oncology grant, equipment sourced and installed, trained staff recruited, and clinical service commenced, ensuring that the long-term vision turned into reality.

The establishment of nuclear medicine at K/T hospital complex has been a significant achievement that will cater to the needs of the North West patients, enhancing diagnostic and therapeutic services by increasing capacity and volume of services rendered, and ultimately improving overall patient outcomes.

Our success has improved referral processes and provided quality services at an affordable cost, reducing the burden on tertiary hospitals in Gauteng. Partnership with Wits Nuclear Medicine in Johannesburg provides flexibility and ease of referral pathway as we continue to expand our range of services.

In conclusion, starting nuclear medicine services in the North West province public health sector has been an overall success story. Our experience demonstrates the importance of foresight, strategic planning and partnerships in turning vision into reality.

By establishing the first nuclear medicine unit in the public health sector of the North West Province, we have improved accessibility and provided a higher quality of care to patients in the region. We hope to inspire similar efforts to expand nuclear medicine services in other relatively underserved regions.

### The South African experience of Re-SCT production

**T. Sello^1^**

^1^ *The South African Nuclear Energy Corporation (Necsa) and NuMeRI*

Rhenium-188 is a beta emitter with a half-life of 17 h and is able to penetrate the human tissue to a depth of 2–3 mm. This makes this therapeutic isotope ideal for the treatment of superficial tissue with no risk to other parts of the body. The relatively short half-life allows treatments to be performed in a very short period of time. Re-SCT is a specialised non-invasive brachytherapy for the treatment of basal and squamous cell carcinomas of the skin. Application of this epidermal radioisotope therapy facilitates local direct cell-killing as a result of the beta-radiation. In this process, both the local death of cells and the local reactions of the immune system of the body to repair itself are triggered.

In partnership with the company currently supplying SCT to several parts of the world, doses were manufactured at Necsa and distributed to various hospitals as part of a market penetration study. The recipient hospitals would have access to the specially designed equipment that is needed to measure and apply the final dose for application. The production of the Re-SCT involved the elution of a Tungsten-188 (W-188) generator with saline into the production unit. Re-188 was processed via a chemical reaction to afford the end product. The paint-based product was transferred into Re-SCT carpoules, which were packaged and sent out for patient treatment. As the first such study in the country, we report the technical challenges and triumphs experienced in the installation of the production set up in our existing infrastructure. Even though a lot of challenges were met, several doses were successfully prepared and used in patient treatment.
